# Lactic acid bacteria modulate the CncC pathway to enhance resistance to β-cypermethrin in the oriental fruit fly

**DOI:** 10.1093/ismejo/wrae058

**Published:** 2024-04-15

**Authors:** Tian Zeng, Qianyan Fu, Fangyi Luo, Jian Dai, Rong Fu, Yixiang Qi, Xiaojuan Deng, Yongyue Lu, Yijuan Xu

**Affiliations:** Guangdong Laboratory for Lingnan Modern Agriculture, Department of Entomology, South China Agricultural University, Guangzhou 510642, China; Guangdong Laboratory for Lingnan Modern Agriculture, Department of Entomology, South China Agricultural University, Guangzhou 510642, China; Guangdong Provincial Sericulture & Mulberry Engineering Research Center, Guangdong Prov Key Lab of AgroAnimal Genomics & Molecular Breeding, College of Animal Science, South China Agricultural University, Guangzhou 510642, China; Guangdong Laboratory for Lingnan Modern Agriculture, Department of Entomology, South China Agricultural University, Guangzhou 510642, China; Guangdong Laboratory for Lingnan Modern Agriculture, Department of Entomology, South China Agricultural University, Guangzhou 510642, China; Guangdong Laboratory for Lingnan Modern Agriculture, Department of Entomology, South China Agricultural University, Guangzhou 510642, China; Guangdong Provincial Sericulture & Mulberry Engineering Research Center, Guangdong Prov Key Lab of AgroAnimal Genomics & Molecular Breeding, College of Animal Science, South China Agricultural University, Guangzhou 510642, China; Guangdong Laboratory for Lingnan Modern Agriculture, Department of Entomology, South China Agricultural University, Guangzhou 510642, China; Guangdong Laboratory for Lingnan Modern Agriculture, Department of Entomology, South China Agricultural University, Guangzhou 510642, China

**Keywords:** symbiotic bacteria, Bactrocera dorsalis, intestinal immunity, cytochrome P450, detoxification

## Abstract

The gut microbiota of insects has been shown to regulate host detoxification enzymes. However, the potential regulatory mechanisms involved remain unknown. Here, we report that gut bacteria increase insecticide resistance by activating the cap “n” collar isoform-C (CncC) pathway through enzymatically generated reactive oxygen species (ROS) in *Bactrocera dorsalis.* We demonstrated that *Enterococcus casseliflavus* and *Lactococcus lactis*, two lactic acid-producing bacteria, increase the resistance of *B. dorsalis* to β-cypermethrin by regulating cytochrome P450 (P450) enzymes and α-glutathione S-transferase (GST) activities. These gut symbionts also induced the expression of CncC and muscle aponeurosis fibromatosis. *BdCncC* knockdown led to a decrease in resistance caused by gut bacteria. Ingestion of the ROS scavenger vitamin C in resistant strain affected the expression of *BdCncC*/*BdKeap1*/*BdMafK*, resulting in reduced P450 and GST activity. Furthermore, feeding with *E. casseliflavus* or *L. lactis* showed that *BdNOX5* increased ROS production, and *BdNOX5* knockdown affected the expression of the *BdCncC*/*BdMafK* pathway and detoxification genes. Moreover, lactic acid feeding activated the ROS-associated regulation of P450 and GST activity. Collectively, our findings indicate that symbiotic gut bacteria modulate intestinal detoxification pathways by affecting physiological biochemistry, thus providing new insights into the involvement of insect gut microbes in the development of insecticide resistance.

## Graphical Abstract

**Graphical Abstract:**
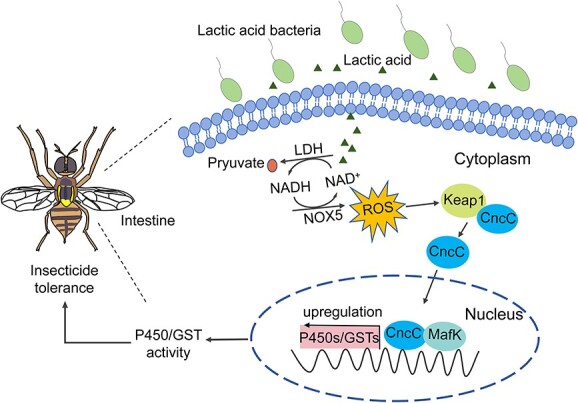

## Introduction

The intricate relationship between insects and their gut microbiota is an extensively studied research topic, underscoring a crucial aspect of insect physiology that influences metabolic, developmental, and immune responses [1–3]. Among these roles, the contribution of the gut microbiota to detoxification processes is important, providing critical insights into insect survival strategies after exposure to plant-derived toxins and synthetic pesticides [4–8]. Additionally, various symbiotic bacteria are involved in the detoxification activity of their hosts. For example, the expression of toxin-degrading enzymes such as cytochrome P450 (P450) enzymes and α-glutathione S-transferase (GST) is induced by the gut microbiota in *Amplicephalus curtulus* [9], *Bombyx mori* [10], and *Anopheles stephensi* [11]. Therefore, symbiotic gut bacteria may also play a crucial role in increasing insecticide resistance by indirectly modulating the activity of detoxifying enzymes in the host. However, the molecular mechanisms governing these processes remain unclear.

Insecticide resistance is a complex process that involves intricate regulatory pathways. Among these transcription factors, cap “n” collar isoform-C (CncC), which is analogous to mammalian Nrf2, has an important role. CncC and its heterodimer partner, muscle aponeurosis fibromatosis (Maf), regulate the overexpression of detoxification enzymes, transporters involved in insecticide resistance, and elements involved in the antioxidant response to xenobiotics [12, 13]. *CncC* gene was overexpressed in insecticide-resistant strains (RSs) of *Drosophila melanogaster.* Conversely, the silencing of *CncC* repressed the expression of several detoxification genes, including *Cyp6g1*, *Cyp12d1*, *Cyp6a2*, *GstD2*, *Cyp6a8*, and *CYP6AB12* [14, 15]. Keach-like ECH-related protein 1 (Keap1) contains cysteine residues that are sensitive to redox reactions and can chelate CncC under low levels of reactive oxygen species (ROS) [15, 16]. However, under ROS-related oxidative stress, CncC dissociates from Keap1, translocates to the nucleus, and binds to Maf [12, 16]. Furthermore, intestinal commensal bacteria, especially *Lactobacillus*, stimulate the enzymatic production of ROS in epithelial cells to promote cell proliferation [17, 18], accelerate recovery from injury [19], and alter epithelial NF-κB signaling [20]. This activation of the Nrf2 pathway has salutary effects against exogenous insults to the intestinal epithelium [21].

*Bactrocera dorsalis* is a prime example of how these mechanisms are important in agricultural contexts [22, 23]. Given that *B. dorsalis* is a major pest that adversely affects diverse fruit crops, elucidating the interactions of *B. dorsalis* with its gut microbiota, particularly how these interactions regulate detoxification pathways and contribute to insecticide resistance, is important for developing effective pest management strategies [6, 24–31]. Previous studies have demonstrated that changes in the microbiota composition, such as increases in *Acetobacter* spp. and *Lactobacillus*, correlate with insecticide resistance, highlighting the role of gut bacteria in modulating host defense mechanisms against these chemical stressors [32]. Nevertheless, the intricacies of how gut commensal bacteria affect the CncC signaling pathway and its downstream effects on detoxification and resistance remain to be fully elucidated.

In this study, we investigated the molecular mechanisms underlying how the gut microbiota regulates insecticide resistance in *B. dorsalis*, focusing on the activation of the *CncC* pathway by ROS enzymatically produced by gut bacteria. We specifically explored how *Enterococcus casseliflavus* and *Lactococcus lactis*, notable lactic acid (LA) producers, influence *β-cypermethrin* resistance, impact *P450* and *GST* activities, and modulate *CncC* pathway gene expression. Through an integrative approach that includes *BdCncC* knockdown and ROS scavenging, we aimed to unravel the complex interactions governing these processes, offering new insights into the dynamic interplay between the gut microbiota and host detoxification mechanisms. This research not only advances our understanding of the biochemical pathways essential for insecticide resistance but also paves the way for innovative pest management strategies that exploit these microbiota-host interactions.

## Materials and methods

### Insect strains and rearing

*B. dorsalis* susceptible strain (SS) was collected from a field in Qingyuan, Guangdong Province, China, in August 2003 and reared indoors for more than 100 generations without exposure to pesticides. RS of *B. dorsalis* was obtained by selection after adult exposure to a β-cypermethrin-treated surface over the course of 40 generations.

*B. dorsalis* flies were reared at 27 ± 1°C with 75 ± 1% relative humidity under a 14:10-h light:dark photoperiod cycle. Hatched larvae were maintained on an artificial diet mainly consisting of bananas before pupation. Pupae were kept in a plastic bucket with wet sand until adults emerged. Adults were fed an artificial diet consisting of yeast extract and dry sugar mixed at a 1:1 ratio (w/w) [33] and housed in transparent plastic cages.

### Bioassay and synergism experiment

The toxicity of β-cypermethrin to different strains was determined with the aid of an 8% sucrose solution with gradient concentrations of the insecticide. Twenty flies of each strain were fed each concentration. Mortality was recorded after 1 day. Three replicates were prepared for each concentration. Five concentrations of insecticide were used to establish the log-probit lines.

With the abovementioned bioassay, a synergistic experiment was conducted with the P450 inhibitor piperonyl butoxide (PBO) and the GST inhibitor diethyl maleate (DEM). PBO (BP1176, Sigma–Aldrich) and DEM (D97703, Sigma–Aldrich) were dissolved in acetone to final concentrations of 10 mM (PBO) and 100 mg/l (DEM), respectively. A 2 μl solution of the inhibitor was topically applied to the pronotum, with acetone as the control. After 2 h, the fruit flies treated with PBO or DEM were transferred to a sugar water solution containing different doses of β-cypermethrin for toxicological experiments. The toxicity ratio was calculated by dividing the median lethal concentration (LC_50_) value of β-cypermethrin alone by the LC_50_ of β-cypermethrin with PBO or DEM [34].

### RNA preparation and transcriptomic sequencing

Total RNA was extracted from the guts of eight 10-day-old adult SS and RS flies (females:males = 1:1) as one biological replicate by using TRIzol reagent (Life Technologies, Carlsbad, CA) according to the manufacturer’s protocol. Three biological replicates were analyzed. The integrity of the RNA was verified by 1% RNase-free agarose gel electrophoresis, and the RNA concentration was determined by measuring the absorbance of the sample at 260 nm using a spectrophotometer (Thermo NanoDrop 2000c; Santa Clara, CA). After the total RNA was extracted, eukaryotic mRNA was enriched with oligo(dT) beads. Then, the enriched mRNA was fragmented into short fragments using fragmentation buffer and reverse transcribed to cDNA by using the NE Next Ultra RNA Library Prep Kit for Illumina (NEB #7530, New England Biolabs, Ipswich, MA). The purified double-stranded cDNA fragments were end repaired, A bases were added, and the fragments were ligated to Illumina sequencing adapters. The ligation reaction mixture was purified with AMPure XP Beads (1.0X). Ligated fragments were subjected to size selection by agarose gel electrophoresis and polymerase chain reaction (PCR) amplification. The resulting complementary DNA (cDNA) library was sequenced on NovaSeq 6000 (Illumina). The raw reads were deposited into the National Center for Biotechnology Information (NCBI) Sequence Read Archive (SRA) database (PRJNA1027602).

### cDNA synthesis and reverse transcription quantitative PCR

RNA was extracted using TRIzol reagent, and the RNA integrity was determined via electrophoresis on a formaldehyde agarose gel. RNA aliquots (1 μg) were reverse transcribed to cDNA using a PrimeScript RT reagent Kit with gDNA Eraser (TaKaRa Bio, Otsu, Japan) according to the manufacturer’s instructions. The biosynthesized cDNA was used as a template for reverse transcription quantitative PCR (RT–qPCR), which was conducted on a CFX Connect Real-Time PCR System (Bio-Rad, Hercules, CA) with TB Green Premix Ex *Taq* II (Tli RNase H Plus) (TaKaRa Bio, Otsu, Japan) and primers designed using the NCBI database (Table S1). The thermal cycling conditions were as follows: 95°C for 30 s, followed by 40 cycles of 95°C for 5 s and 60°C for 34 s. The transcript levels of different genes were quantified using the 2^−ΔΔCT^ method. *α-Tubulin* and *RPL32* [35] were used as reference genes for gene expression analysis in *B. dorsalis* due to their stable expression.

### P450 and GST activity assays

The p-nitrophenyl ether-O-demethylase activity of P450 in the gut of *B. dorsalis* was measured by using p-nitroanisole (p-NA) as a substrate, as previously described [15]. The midguts of 20 adult flies were dissected in ice-cold Phosphate Buffer Saline (PBS) and homogenized with 200 μl of 0.1 M PBS (pH = 7.5) containing 1 mM ethylenediaminetetraacetic acid, 1 mM phenylmethanesulfonyl fluoride, 1 mM phenylthiourea, 1 mM dithiothreitol, and 10% glycerol. The supernatant was then centrifuged at 4°C for 20 min at 12000 rpm to prepare an enzyme stock solution. Twenty microliters of the supernatant were added to PBS containing 0.2 μM p-NA and 9.6 mM nicotinamide adenine dinucleotide phosphate (NADPH) for the enzyme reaction. After incubation at 30°C for 30 min, the absorbance at a wavelength of 405 nm was measured using a microplate reader. The P450 activity in the supernatant was calculated by creating a standard curve using the A405 values for different concentrations of p-nitrophenol. The protein concentration in the enzyme supernatant was measured using a Bovine Serum Albumin (BSA) assay kit (Sangon Biotech, Shanghai, China).

A GST activity assay kit (Sangon Biotech, Shanghai, China) was used to measure GST activity in the gut of *B. dorsalis*. GST catalyzes the binding of glutathione to 1-chloro-2,4-dinitrobenzene, and the binding product has an optical absorption peak at 340 nm. GST activity can be calculated by measuring the rate of increase in the absorbance at 340 nm. We dissected the midguts of 20 adult flies in ice-cold PBS and then homogenized them with 200 μl of Reagent I (available in the reagent kit). Next, the supernatant was obtained by centrifugation at 12000 rpm at 4°C for 20 min. The enzymatic reaction was carried out according to the kit manufacturer’s instructions, and then, the GST activity of the supernatant was calculated from the protein concentration.

### Antibiotic treatment of *B. dorsalis* and oral ingestion of bacteria

For the RS + axenic flies, 5-day-old RS *B. dorsalis* adults were fed 8% sugar water supplemented with 50 μg/ml tetracycline, 100 μg/ml penicillin–streptomycin, 150 μg/ml gentamycin, and 150 μg/ml rifampicin for 4 days. Then, the flies were treated with sterile water for 1 day to metabolize the antibiotics. Before dissection, the flies were sterilized by soaking in 70% alcohol for 3 min and then washed three times with sterile PBS. We dissected the intestines in sterile PBS. PCR using universal 16S rDNA gene primers (Table S1) and colony-forming unit (CFU) assays was used to verify the bactericidal efficacy of antibiotics. Then, the guts were dissected to extract DNA and RNA, and P450 and GST activities were measured.

For the RS + axenic+bacteria group, *E. casseliflavus*, *L. lactis*, *Klebsiella pneumoniae*, and *Enterobacter hormaechei* were isolated from the gut of the RS flies. The bacteria were grown in de Man–Rogosa–Sharpe medium at 37°C and 200 rpm for 16 h. The bacterial culture was then pelleted by centrifugation (3000 × *g*, 15 min), washed twice in sterile PBS, and resuspended in 8% sterile sucrose solution. The RS axenic flies were fed a bacterial suspension (OD600 = 5) for 3 days. The SS flies were fed different concentrations of bacterial suspension (OD600 = 0.5, 5, and 10) for 3 days. Then, the guts were dissected to extract DNA and RNA, and the P450 and GST activities were measured.

### Gut bacterial DNA sample preparation and 16S rRNA gene amplicon sequencing

The guts of 16 SS or RS flies were dissected under sterile conditions, and DNA was extracted using an E.Z.N.A. Soil DNA kit (Omega Bio-Tek, Inc., Norcross, GA) according to the manufacturer’s instructions. Five biological replicates per strain were analyzed. The 16S rRNA gene V3–V4 region was amplified by the primers 341-F (5′-CCTACGGGNGGCWGCAG-3′) and 806-R (5′ –GGACT ACHVGGGTATCTAAT-3′). The PCR products were assessed using 2% agarose gel electrophoresis, purified using an AxyPrep DNA gel extraction kit (Axygen Biosciences, Union City, CA), and quantified using an ABI StepOnePlus Real-Time PCR System (Life Technologies, Foster City, USA). The purified amplicons were pooled in equimolar amounts and paired-end sequenced (PE250) on NovaSeq 6000 (Illumina) according to the standard protocols. The raw reads were deposited into the NCBI SRA database (PRJNA1027561).

### Quantification of specific gut bacteria by quantitative PCR

The loads of *E. casseliflavus* and *L. lactis* in the gut were quantified by real-time PCR using specific primers and normalized to real-time PCR data for the host *β-actin* gene [36]. The primer pairs used in the quantitative PCR analysis are shown in Table S1. Genomic DNA from the intestinal tracts of *B. dorsalis* was extracted using an EZNA Soil DNA Kit (Omega Bio-Tek, Inc., Norcross, GA) according to the manufacturer’s instructions.

### Prediction of the binding site for the transcription factors CncC–Maf

The promoter sequence information for detoxification genes (P450s and GSTs) downregulated in the RS flies after elimination of gut bacteria was obtained from the *B. dorsalis* genome. A 2000 bp fragment upstream of the transcription start site of each gene was selected as a potential promoter region for prediction of the CncC–Maf binding site by JASPAR [37].

### Dual-luciferase reporter gene assay

The open reading frame sequence of *BdCncC* was amplified using cDNA from *B. dorsalis* as a template with the primers specified in Table S1. The *BdCncC* overexpression vector was constructed with the BsmBI and Esp3I restriction enzyme sites on the pcDNA3.1 plasmid. Additionally, the promoter regions of the P450 and GST genes were amplified from *B. dorsalis* DNA using primers from Table S1 and further utilized to construct promoter vectors on the pGL3-basic plasmid with SacI and HindIII restriction enzyme sites. The internal reference plasmid used was pGL4-TK.

HEK-293 T cells were cultured, and 1 day prior to transfection, the cells were plated in a 48-well plate. Transfection was carried out using Lipofectamine 3000 (L3000015, Invitrogen) reagent; cotransfection of pcDNA3.1(+)BdCncC, pGL3-basic-promoter, and pGL4-TK plasmids at a ratio of 1:1 for the promoter plasmid to the *BdCncC* overexpression plasmid was performed; and transfection of pGL4-TK, which represented one-tenth of the total system, was performed. Control transfections included the pcDNA3.1, pGL3-basic-promoter, and pGL4-TK plasmids. After 48 h of transfection, the Dual-Luciferase Reporter Assay System (E1910, Promega) was used to measure firefly and renilla luciferase activities. Cell lysis, reagent addition, and readings were conducted according to the manufacturer’s instructions. The assay included three biological replicates, and values were recorded for the subsequent analysis.

### dsRNA-mediated gene silencing

DNA templates recovered from PCR amplification using primers (Table S1) containing T7 promoter sequences at the 3′ and 5′ ends were used to biosynthesize *BdCncC*, *BdNOX5*, *BdLDH*, *LOC105222599-CYP6g1*, *LOC105233823-CYP6g1*, *LOC105222603-CYP6g1*, *LOC105226935-CYP6v1*, *LOC105226035-CYP6d4*, *LOC105232220-CYP4ae1*, *LOC105225813-GSTD1*, *LOC105229682-GSTT1*, *LOC105222427-GSTD7*, and *GFP* (negative control) dsRNA. A MEGAscript T7 transcription kit (Ambion, Austin, TX) was used to produce the specific dsRNA of each gene following the manufacturer’s instructions. Two micrograms of dsRNA were injected into the thoracic hemocoel of *B. dorsalis* adults using a FemtoJet microinjection system (Eppendorf).

### Vitamin C treatment

Vitamin C (VC) is an antioxidant, and in previous studies, feeding VC to *B. dorsalis* effectively reduced ROS levels in the gut [36]. Ten-day-old adult flies of RS *B. dorsalis* were fed 8% sugar water containing 100 mg/ml VC (PHR1008, Sigma–Aldrich) for 24 h. Flies fed 8% sugar water were used as the controls. Then, the dissected guts were subjected to P450 and GST activity analysis, ROS activity assays, and RT–qPCR.

### Analysis of ROS activity *in vivo*

We used a hydrogen peroxide assay kit (Beyotime Biotechnology, Shanghai, China) to measure the production of H_2_O_2_ and determine the activity of ROS with the ROS fluorescent probe dihydroethidium (DHE; D7008, Sigma–Aldrich). The midguts were then dissected in cold PBS containing 2 mg/ml of the catalase inhibitor 3-amino-1,2,4-triazole (A8056, Sigma–Aldrich). Twenty dissected guts (females:males = 1:1) were combined into a sample and homogenized in lysis buffer to measure the concentration of H_2_O_2_ [38]. Other guts were immediately incubated in 2 μM DHE in PBS to assess ROS activity [38].

### Measurement of lactate content

The amount of lactate in dissected guts (*n* = 20, same number of males and females) was determined using a Lactate Assay Kit (Sangon Biotech, Shanghai, China) following the manufacturer’s instructions and was normalized to the total protein content.

### Measurement of pyruvate content

A pyruvate (PA) content detection kit (Sangon Biotech, Shanghai, China) was used to measure the PA content in the gut of *B. dorsalis*. PA plays an important pivotal role in linking glucose, fatty acids, and amino acids through acetyl-CoA metabolism. Acetate reacts with 2,4-dinitrophenylhydrazine to produce PA-2,4-dinitrophenylhydrazone, which appears cherry red in alkaline solution. PA in a sample can be detected at a wavelength of 520 nm. Twenty intestines were placed in 200 μl of extraction buffer (provided by the reagent kit) for ice-cold homogenization. The mixture was allowed to stand for 30 min, followed by centrifugation at 8000 × *g* for 10 min at room temperature. The supernatant was then collected for subsequent analysis following the manufacturer’s instructions.

### LA feeding

SS and axenic RS adult flies (with the same number of males and females) were fed DL-LA (69 785, Sigma–Aldrich). The axenic RS flies were fed 50 μM DL-LA for 3 days. The SS flies were fed different concentrations of DL-LA (5 μM, 50 μM, 500 μM, 5 mM, or 50 mM) for 2 days. Then, the dissected guts were subjected to P450 and GST activity assays, ROS activity assays and RT–qPCR.

### Statistical analysis

The mortality data of the flies were corrected using Abbott’s formula [39], and the LC_50_ values (50% killing concentration of flies) and 95% fiducial limits of the LC_50_ for each strain were determined via probit analysis using Statistical Package for the Social Sciences 27.0 software. Two LC_50_ values were considered significantly different if their 95% fiducial limits did not overlap [40]. Other statistical analyses were performed using Prism 7 (GraphPad Software). Two-tailed independent *t*-tests were used for unpaired comparisons between two groups of data. For comparisons of three or more sets of data, one-way analysis of variance (ANOVA) was performed, followed by Tukey’s multiple comparison test. A value of *P* < .05 was considered indicative of statistical significance.

## Results

### Gut symbiotic bacteria regulate resistance to β-cypermethrin in *B. dorsalis*

**Figure 1:**
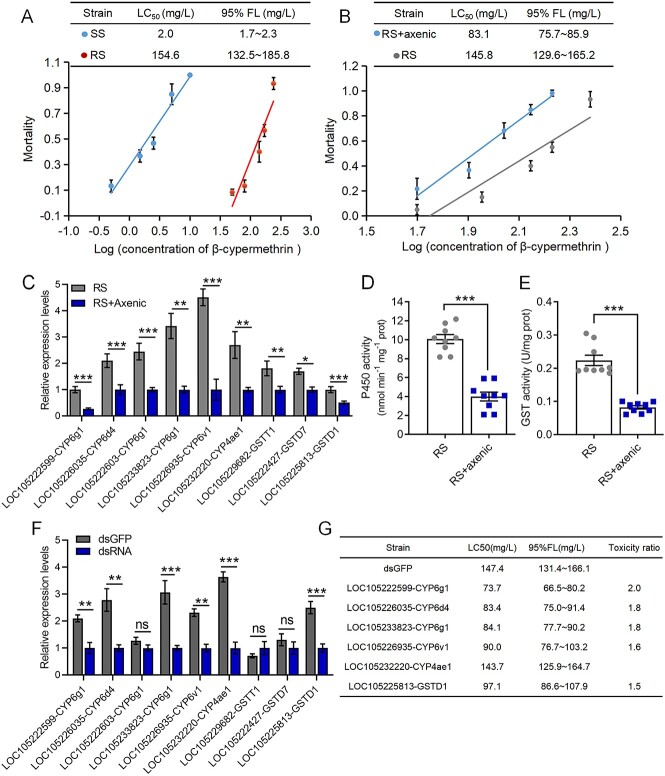
The elimination of symbiotic gut bacteria reduces resistance to β-cypermethrin in *B. dorsalis*; (A) susceptibility of the SS and RS flies to β-cypermethrin; LC_50_, lethal concentration that killed 50% of *B. dorsalis* adults; 95% FL, 95% fiducial limit of the LC_50_; (B) susceptibility of the RS and RS + axenic flies to β-cypermethrin; (C) gut transcriptional responses of *LOC105222599-CYP6g1*, *LOC105233823-CYP6g1*, *LOC105222603-CYP6g1*, *LOC105226035-CYP6d4*, *LOC105226935-CYP6v1*, *LOC105232220-CYP4ae1*, *LOC105225813-GSTD1*, *LOC105229682-GSTT1*, and *LOC105222427-GSTD7* to the elimination of gut symbiotic bacteria in the RS flies; (D) P450 activity and (E) GST activity in the gut of *B. dorsalis* between the RS and RS + axenic strain flies; (F) the interference efficiency of P450 and GST dsRNA injection after 48 h; (G) changes in the LC_50_ of β-cypermethrin after P450 and GST gene interference; the toxicity ratio was calculated by dividing the LC_50_ of ds-*GFP* by that of ds-P450s or ds-GSTs; LC_50_ values were considered significantly different if their fiducial limits did not overlap; Student’s *t*-test was performed for C–F; error bars indicate ± s.e.m.; ^***^P < .001, ^**^P < .01, ^*^P < .05, ns indicates *P* > .05; all results were obtained from at least two independent experiments.

To confirm the resistance level of the *B. dorsalis* SS and RS flies, we examined the toxicity of ingested β-cypermethrin. The LC_50_ values of β-cypermethrin for the SS and RS flies were 2.0 mg/l and 154.6 mg/l, respectively (Fig. 1A). Moreover, the results of both intestinal transcriptomics and real-time fluorescence quantitative PCR showed that the expression of several cytochrome P450 genes and GST genes was significantly greater in the RS flies than in the SS flies (Fig. S1A–C). The corresponding P450 enzyme activity and GST enzyme activity of the RS flies were also significantly greater than those of the SS flies (Fig. S1D and E). The P450 inhibitor PBO and the GST inhibitor DEM reduced the LC_50_ value of β-cypermethrin in the RS flies from 151.8 mg/l to 67.9 mg/l (PBO) and 77.5 mg/l (DEM), respectively, with toxicity ratios of 2.2 and 2.0, respectively (Fig. S2).

After the elimination of intestinal symbiotic bacteria (Fig. S3A), the LC_50_ value of β-cypermethrin for the RS flies decreased from 145.8 mg/l to 83.1 mg/l (Fig. 1B). After antibiotic treatment, the expression of the P450 and GST genes in the gut was significantly reduced in the RS flies, decreasing by 7.7%–70.8% (Fig. 1C). However, no difference was found in the expression of other detoxification genes (Fig. S3B). Moreover, the intestinal P450 activity and GST activity decreased by 60.4% and 63.2%, respectively, in the RS flies after antibiotic treatment (Fig. 1D and E).

To validate the functionality of these detoxification genes, we successfully interfered with the expression of the P450 and GST genes, except *LOC105222603-CYP6g1*, *LOC105229682-GSTT1*, and *LOC105222427-GSTD7* (Fig. 1F). Compared with those in the ds-*GFP* strain, the LC_50_ values for β-cypermethrin were significantly lower in the *LOC105222599-CYP6g1* (2.0-fold), *LOC105226035-CYP6d4* (1.8-fold), *LOC10523823-CYP6g1* (1.8-fold), *LOC105226935-CYP6v1* (1.6-fold), and *LOC105225813-GSTD1* (1.5-fold) knockdown strains (Fig. 1G). However, ds-*LOC105232220-CYP4ae1* did not induce a significant change in the LC_50_ (Fig. 1G). Our experimental findings indicate that the decreased resistance of *B. dorsalis* to β-cypermethrin following gut microbiota depletion was due to the downregulation of the P450 and GST genes.

### Differences in the gut microbiota community structure between the RS and SS flies

To explore the role of the gut microbiota in regulating insecticide resistance, we conducted 16S rDNA sequencing analysis of the gut microbiota of the SS and RS flies. We obtained a total of 12 sets of experimental data, and PCA based on weighted UniFrac distances revealed differences in the gut bacterial community structure between the SS and RS flies (Fig. S4A). The Shannon index, Pielou index, and Simpson index of the gut microbiota in the RS flies were significantly different from those in the SS flies (Fig. S4B–D).

**Figure 2:**
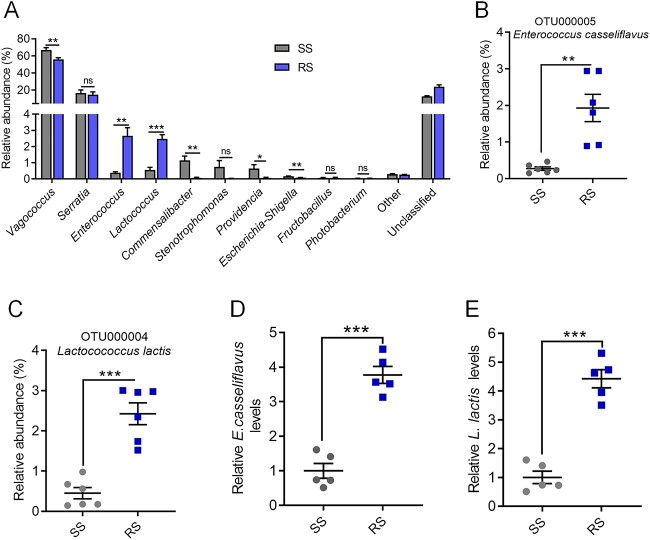
Differences in gut symbiotic bacteria between the SS and RS of *B. dorsalis*; (A) relative genus-level abundance profiles of bacteria in the guts of the SS and RS flies; relative abundances of the species (B) *E. casseliflavus* and (C) *L. lactis* in the SS and RS flies, as determined by 16S rDNA sequencing; the loads of (D) *E. casseliflavus* and (E) *L. lactis* in the guts of the SS and RS flies; the housekeeping gene β-actin was used as an endogenous control in D and E; Student’s *t*-test was performed for A–E; error bars indicate ± s.e.m.; ^***^P < .001, ^**^P < .01, ^*^P < .05; all results were obtained from at least two independent experiments.

In a comparison of the composition and structure of gut bacterial communities between the RS and SS flies, we observed significant changes in the abundances of multiple gut bacteria in the RS flies. Specifically, at the genus level, the abundances of the intestinal probiotics *Enterococcus* and *Lactococcus* were significantly increased by 7.0-fold and 4.4-fold, respectively, whereas those of *Vagococus*, *Commesalibacter*, *Stenotrophomonas*, *Escherichia Shigella*, and *Providencia* were significantly reduced (Fig. 2A). Furthermore, at the species level, we also found a significant increase in the abundances of *E. casseliflavus* (OTU000005) and *L. lactis* (OTU000004) (Fig. 2B and C and Fig. S4E). Moreover, the fluorescence quantitative PCR results of the 16S rRNA gene showed that the relative abundances of *E. casseliflavus* and *L. lactis* in the RS flies were significantly greater than those in the SS flies (Fig. 2D and E). Therefore, we speculated that these two bacteria play crucial roles in the development of β-cypermethrin resistance.

### *L. lactis* and *E. casseliflavus* enhance resistance to β-cypermethrin in *B. dorsalis*

**Figure 3:**
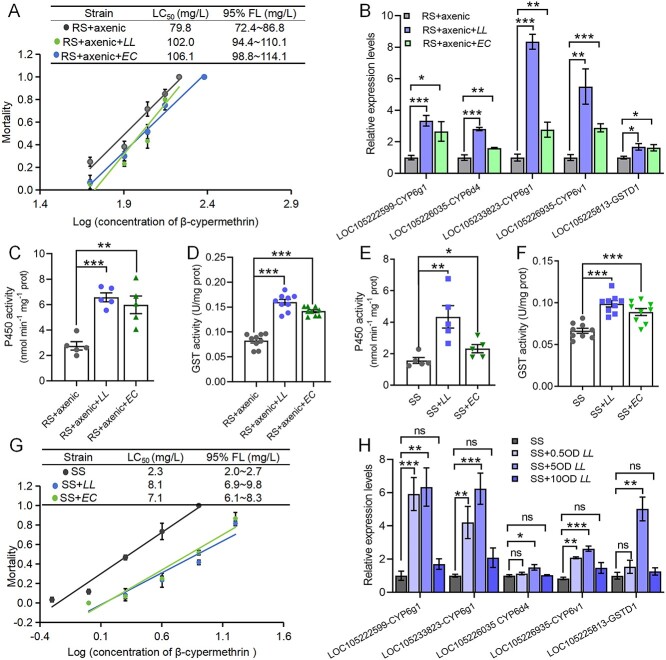
Intestinal symbiotic bacteria increase resistance to β-cypermethrin in *B. dorsalis*; (A) toxicity regression analyses of the RS + axenic, RS + axenic+*EC*, and RS + axenic+*LL* fly responses to β-cypermethrin; (B) gut transcriptional responses of *LOC105222599-CYP6g1*, *LOC105233823-CYP6g1*, *LOC105226035-CYP6d4*, *LOC105226935-CYP6v1*, and *LOC105225813-GSTD7* to elimination of gut symbiotic bacteria followed by separate feeding of the RS flies with *E. casseliflavus* and *L. lactis*; (C) P450 activity and (D) GST activity in the gut of the RS + axenic, RS *+* axenic*+LL* and RS + axenic*+EC* strain flies; (E) P450 activity and (F) GST activity in the gut of the SS, SS *+ LL*, and SS *+ EC* strain flies; (G) susceptibility response to β-cypermethrin in the SS, SS *+ LL*, and SS *+ EC* strain flies; LC_50_ values were considered significantly different if their fiducial limits did not overlap; (H) gut transcriptional responses of detoxification genes in the SS flies after feeding different concentrations (0.5 OD, 5 OD, and 10 OD) of *L. lactis* (*LL*) for 3 days; RS + axenic strain: RS strain fed mixed antibiotic and sugar water solution for 4 days; RS + axenic+*LL/EC* strain: RS + axenic strain flies fed aseptic water for 24 h followed by continuous feeding of a 5 OD bacterial solution of *L. lactis* or *E. casseliflavus* for 3 days; SS *+ LL/EC*: SS flies continuously fed 5 OD *L. lactis* (*E. Casseliflavus*) for 3 days; Student’s *t*-test was performed for B–F, and one-way ANOVA followed by Tukey’s multiple comparison test was performed for H; error bars indicate ± s.e.m.; ^***^P < .001, ^**^P < .01, ^*^P < .05, ns indicate *P* > .05; all results were obtained from at least two independent experiments.

We further isolated *E. casseliflavus* and *L. lactis* from the gut of the RS flies and orally fed them to the axenic RS flies (Fig. S5A and B). Toxicity tests revealed that both *E. casseliflavus* and *L. lactis* reversed the antibiotic-induced decreases in β-cypermethrin resistance, with the LC_50_ values increasing from 79.8 mg/l to 106.1 mg/l (*E. casseliflavus*) and 102.2 mg/l (*L. lactis*) (Fig. 3A). However, after oral administration of the intestinal bacteria *K. pneumoniae* and *E. hormaechei* (Fig. S6A), the LC_50_ of β-cypermethrin did not significantly increase in the axenic RS flies (Fig. S6B). The P450 and GST genes were significantly activated by *E. casseliflavus* and *L. lactis* (Fig. 3B). The intestinal P450 and GST activities were significantly greater in these flies than in those treated with antibiotics (Fig. 3C and D). To determine whether gut microbes exert a similar impact on SS flies, we fed SS flies with 5 OD *E. casseliflavus* or *L. lactis* (Fig. S5C and D). Similar to the observations in the RS flies, *E. casseliflavus* and *L. lactis* significantly elevated the P450 and GST activities (Fig. 3E and F). The LC_50_ of β-cypermethrin for the SS flies fed *E. casseliflavus* and *L. lactis* increased from 2.3 mg/l to 7.1 mg/l and 8.1 mg/l, respectively (Fig. 3G).

Given that *E. casseliflavus* and *L. lactis* similarly increase insecticide resistance, we used *L. lactis* for gradient feeding experiments in SS flies to investigate the role of the abundance of symbiotic bacteria in the gut microbiota in the development of insecticide resistance. Feeding *L. lactis* at 0.5 OD and 5 OD induced the expression of intestinal P450 and GST genes (Fig. 3H), confirming that increasing the abundance of *L. lactis* in the intestine of susceptible flies promoted insecticide resistance. However, feeding at 10 OD did not induce the expression of detoxification genes (Fig. 3H). Collectively, our data suggest that increases in the abundances of *E. casseliflavus* and *L. lactis* in the fly guts increase their resistance to β-cypermethrin by stimulating P450 and GST activity.

### The gut microbiota regulates resistance to β-cypermethrin by activating the CncC pathway

**Figure 4:**
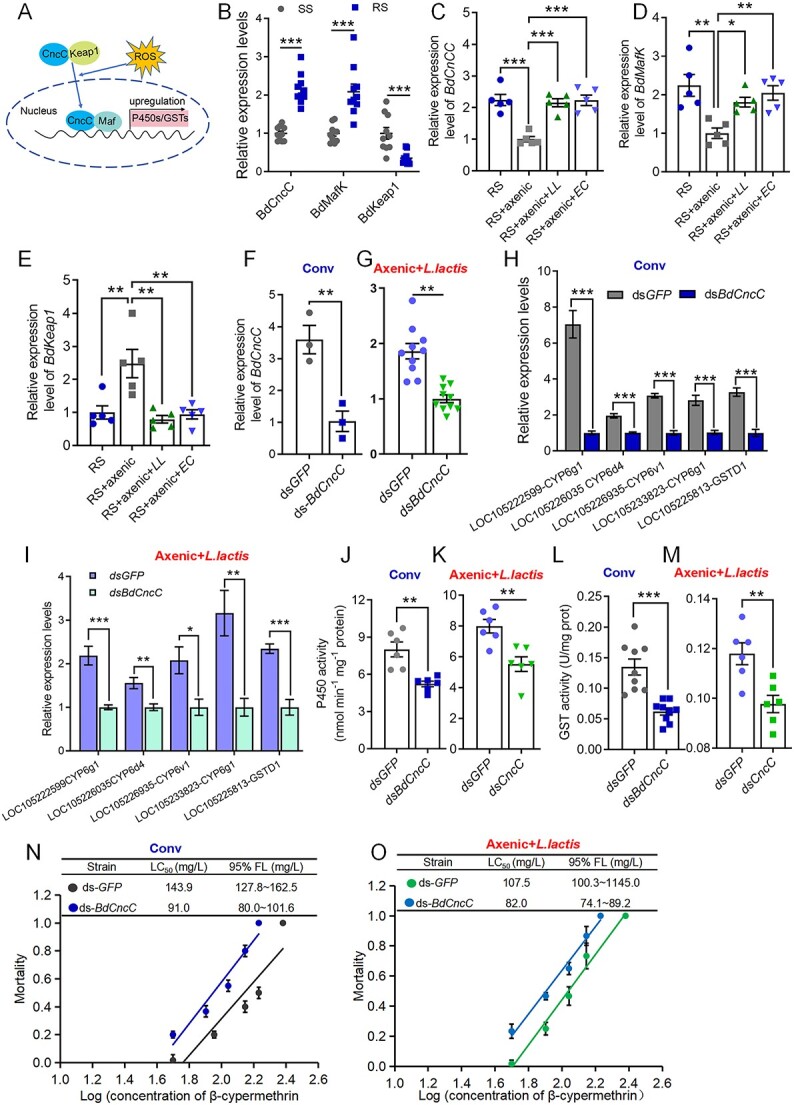
Gut symbiotic bacteria regulate β-cypermethrin resistance in *B. dorsalis* by activating the CncC pathway; (A) CncC/Keap1/Maf pathway regulatory model in insects; (B) quantitative PCR of the *BdCncC*, *BdMafK*, and *BdKeap1* genes in the intestines of the SS and RS flies; gut transcriptional response of (C) *BdCncC*, (D) *BdMafK*, and (E) *BdKeap1* in the nonaxenic (RS), RS + axenic, RS + axenic+*LL*, and RS + axenic*+EC* strain flies; gut *BdCncC* silencing efficiency in *B. dorsalis* at 72 h postinjection with 2.0 μg of ds-*GFP* or ds-*BdCncC* in (F) RS and (G) RS + axenic+*LL* strain flies; the data were normalized to the expression levels in the ds-*GFP*-treated flies; gut transcriptional response of LOC105222599-CYP6g1, *LOC105233823-CYP6g1*, *LOC105226035-CYP6d4*, *LOC105226935-CYP6v1*, and *LOC105225813-GSTD1* to gut *BdCncC* silencing at 72 h in (H) the RS and (I) RS + axenic+*LL* strain flies; P450 activity in the gut of (J) the RS and (K) RS + axenic*+ LL* strain flies after *BdCncC* interference for 72 h; GST activity in the gut of (L) the RS and (M) RS + axenic*+LL* strain flies after *BdCncC* interference for 72 h; effect of *BdCncC* knockdown on the response of (N) RS flies and (O) RS + axenic*+LL* flies to β-cypermethrin; RS + axenic strain: RS strain fed mixed antibiotic and sugar water solution for 4 days; RS + axenic+*LL/EC* strain: RS + axenic strain flies fed aseptic water for 24 h followed by continuous feeding of 5 OD bacterial solution of *L. lactis* or *E. casseliflavus* for 3 days; Conv, conventionally colonized flies; Axenic, axenic flies; LC_50_ values were considered significantly different if their fiducial limits did not overlap; Student’s *t*-test was performed for B–M; error bars indicate ± s.e.m.; ^***^P < .001, ^**^P < .01, ^*^P < .05; all results were obtained from at least two independent experiments.

Our previous studies demonstrated that BdCncC belongs to the bZIP superfamily based on its structural domains and is highly expressed in the intestine [33]. Members of the Cnc-bZIP transcription factor superfamily can regulate genes involved in enhancing insecticide metabolic resistance (Fig. 4A). In this context, we evaluated the expression of genes in the CncC pathway in SS and RS flies. We observed significant upregulation of *BdCncC* and *BdMafK* expression in the CncC pathway in the intestine of the RS flies compared to the SS flies (Fig. 4B). Moreover, the expression of *BdKeap1* was significantly downregulated (Fig. 4B). Furthermore, we analyzed the pretranscriptional 2000 bp fragments of the P450 and GST genes in the gut as potential promoter regions for predicting the CncC–Maf binding site of transcription factors. The prediction results revealed the presence of the CncC–Maf binding site in all resistance-related gene promoters. Specifically, the binding site was located within the following ranges: *LOC105222599-CYP6g1* from positions −1721 to −1708, *LOC105226935-CYP6v1* from −1552 to −1538, *LOC105226035-CYP6d4* from −500 to −486, *LOC105233823-CYP6g1* from −1771 to −1757, and *LOC105225813-GSTD1* from −1231 to −1217 (Fig. S7A). Using a dual-luciferase reporter gene assay, we further confirmed that overexpression of *BdCncC* significantly increased the promoter activity of the target P450 and GST genes by 1.5–3.7-fold compared to that of the pcDNA3.1 control (Fig. S7B).

The RT–qPCR results demonstrated a significant reduction in the expression levels of *BdCncC* (Fig. 4C) and *BdMafK* (Fig. 4D) upon elimination of intestinal bacteria in the RS flies, accompanied by a notable increase in the expression level of *BdKeap1* (Fig. 4E). However, supplementation with the gut symbiotic bacteria *L. lactis* or *E. casseliflavus* effectively counteracted the downregulation of *BdCncC* and *BdMafK* expression resulting from elimination of the gut bacteria (Fig. 4C and D). Additionally, this treatment inhibited the increase in *BdKeap1* expression levels (Fig. 4E).

To assess the functionality of *BdCncC*, we silenced the expression of the *BdCncC* gene in traditionally fed RS flies with 71.3% interference efficiency at 72 h (Fig. 4F). Compared to those in the flies treated with RS-*GFP*-RNAi, the expression levels of the P450 and GST genes harboring CncC–Maf binding sites were significantly decreased in the intestines of the flies subjected to RS-*BdCcnC*-RNAi (Fig. 4H). Correspondingly, there was a significant decrease in the enzyme activities of both P450 and GST (Fig. 4J and L). Additionally, in a separate interference experiment in which *BdCncC* was used as an off-target control, similar alterations in the levels of the detoxification enzymes P450 and GST were identified at 72 h (Fig. S8).

To verify that intestinal bacteria-mediated insecticide resistance is dependent on the expression of the *BdCncC* gene, we injected ds-*BdCncC* into RS flies that had been fed *L. lactis* following antibiotic treatment. After *BdCncC* interference, the enzyme activity and expression levels of the associated P450 and GST genes were significantly lower than those in the ds-GFP control group (Fig. 4G, I, K, and M). This observed pattern was consistent with the results of the toxicity assay (Fig. 4N and O). In conclusion, these findings collectively suggest that *L. lactis* increases the resistance of *B. dorsalis* to β-cypermethrin by regulating *BdCncC*, ultimately activating the expression of P450 and GST.

### Gut microbiota-mediated ROS activate the CncC pathway to increase resistance to β-cypermethrin

**Figure 5:**
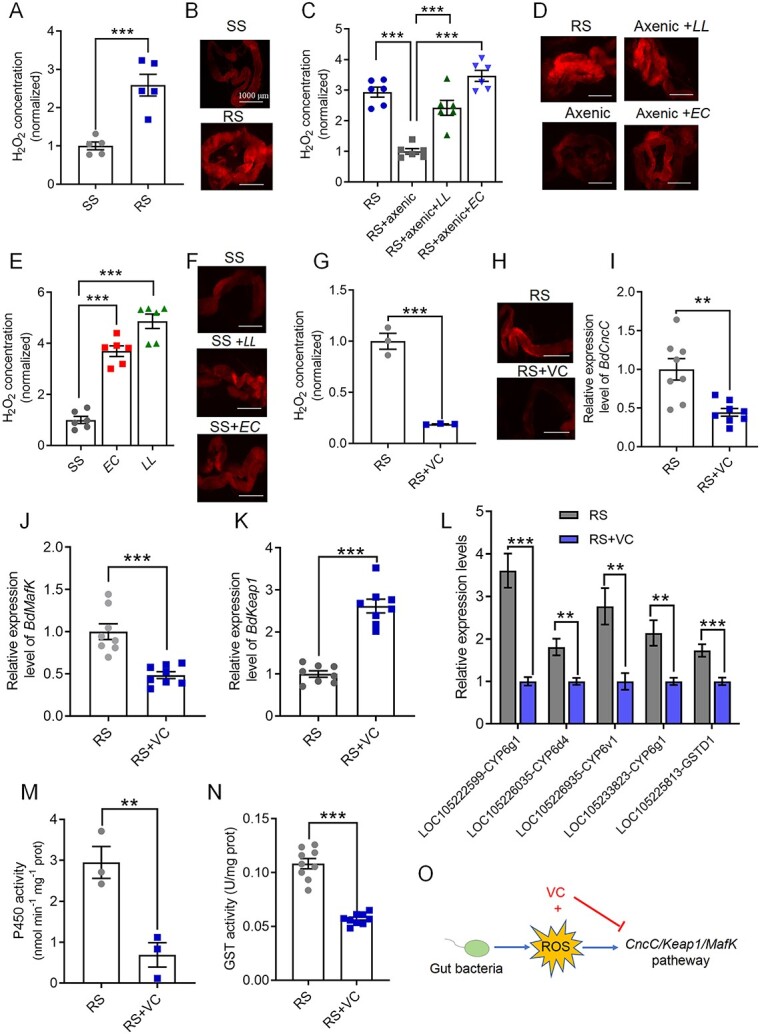
The activation of ROS by intestinal commensal bacteria increases the activity of detoxification enzymes in *B. dorsalis*; ROS activity in the gut of the SS and RS flies was determined by (A) H_2_O_2_ assays and (B) DHE analysis; ROS activity in the gut after elimination of intestinal bacteria and refeeding with *E. casseliflavus* or *L. lactis* in the RS flies was measured by (C) H_2_O_2_ assay and (D) DHE analysis; the ROS activity of the gut after continuous feeding of *E. casseliflavus* or *L. lactis* for 3 days in the SS flies was measured by (E) H_2_O_2_ assays and (F) DHE analysis; ROS activity in the gut after VC treatment in the RS flies was measured by (G) H_2_O_2_ assays and (H) DHE analysis; gut transcriptional response of the (I) *BdCncC*, (J) *BdMafK*, (K) *BdKeap1*, and (L) P450 or GST genes to VC treatment for 24 h in the RS flies; (M) P450 and (N) GST activity in the gut of the RS flies after VC treatment; (O) VC scavenges ROS in the gut and negatively modulates CncC pathway regulation; RS + axenic strain: RS strain fed mixed antibiotic and sugar water solution for 4 days; RS + axenic+*LL/EC* strain: RS + axenic strain flies fed aseptic water for 24 h followed by continuous feeding of a 5 OD bacterial solution of *L. lactis* or *E. casseliflavus* for 3 days; RS + VC: RS flies were fed 8% sugar water containing 100 mg/ml VC for 24 h; SS *+ LL/EC* strain: SS flies continuously fed 5 OD *L. lactis* (*E. casseliflavus*) for 3 days; the H_2_O_2_ levels in A and E were normalized to those in the SS controls, those in C were normalized to those in the RS + axenic flies, and those in G were normalized to those in the RS flies; the scale bars in B, D, F, and H represent 1000 μm; Student’s *t*-test was performed for A, C, E, G, and I–N; error bars indicate ±s.e.m. ^***^P < .001, ^**^P < .01; all results were obtained from at least two independent experiments.

ROS induce the heterodimeric segregation of CncC from Keap1, leading to the translocation of CncC to the nucleus where it heterodimerizes with Maf. This heterodimer binds to and activates the expression of a CncC/Maf response element located in the promoter of the ectopically responsive gene [13] (Fig. 4A). In our study, the H_2_O_2_ content and ROS activity were significantly greater in the gut of the RS flies than in that of the SS flies (Fig. 5A and B). After elimination of the intestinal bacteria, the H_2_O_2_ content in the RS intestine decreased significantly (Fig. 5C), resulting in a corresponding attenuation of ROS activity (Fig. 5D). However, supplementation with *L. lactis* or *E. casseliflavus* individually increased the ROS levels (Fig. 5C and D). Additionally, feeding each of the two bacteria separately to SS flies also stimulated intestinal ROS activity (Fig. 5E and F).

To determine whether the high level of intestinal ROS activity in the gut of the RS flies activated the CncC pathway, we fed RS flies VC, an ROS scavenger. After feeding with VC, the intestinal H_2_O_2_ content of the RS flies decreased, and the ROS activity was attenuated compared with that in the flies fed sugar water (Fig. 5G and H). This reduction in intestinal ROS activity resulted in a significant downregulation of the ROS-regulated transcription factors *BdCncC* and *BdMafK* (Fig. 5I and J) and a corresponding upregulation of *Bdkeap1* expression (Fig. 5K). Moreover, the P450 and GST genes, which are transcriptionally regulated by *BdCncC/BdMafK*, exhibited significant downregulation (Fig. 5L). Consequently, the corresponding ROS scavenging-related P450 and GST enzyme activities were reduced by 76.6% and 47.6%, respectively (Fig. 5M and N). Therefore, we concluded that the gut microbiota of *B. dorsalis* activates the *BdCncC* pathway by producing ROS (Fig. 5O), which in turn regulate insecticide pesticide resistance in *B. dorsalis*.

### *BdNOX5* is required for insecticide resistance

**Figure 6:**
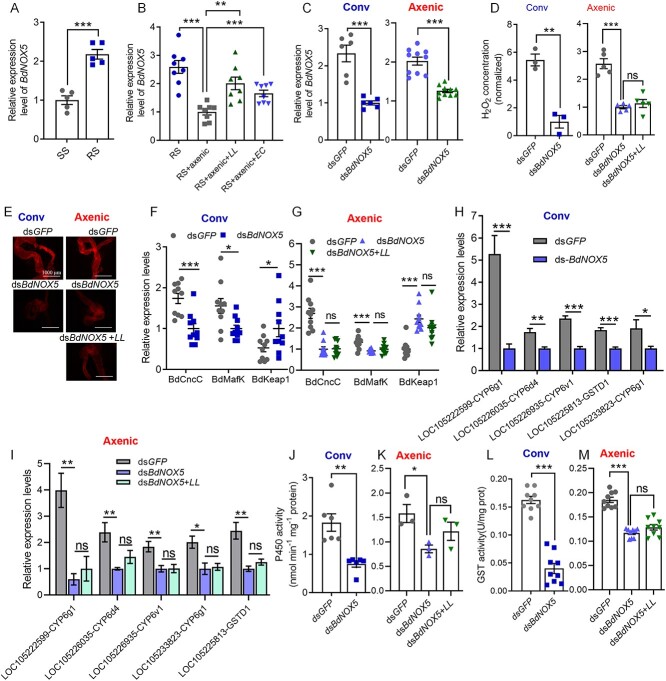
Pesticide resistance in *B. dorsalis* is increased by ROS activation via *BdNOX5* of intestinal commensal bacteria; (A) intestinal *BdNOX*5 gene expression was elevated in the RS group compared to the SS group; (B) gut transcriptional response of *BdNOX5* in the RS, RS + axenic, RS *+* axenic*+LL*, and RS *+* axenic*+EC* strain flies; (C) gut *BdNOX5* silencing efficiency in *B. dorsalis* at 72 h postinjection of 2.0 μg of ds-*GFP* or ds-*BdNOX5* in the RS and RS + axenic strain flies; the data were normalized to the expression levels in the ds-*BdNOX5*-treated flies; the effects of BdNOX5 silencing on ROS activity in the gut of the RS and RS + axenic strain flies were measured by (D) H_2_O_2_ assays and (E) DHE analysis; the H_2_O_2_ levels in D were normalized to those of the ds-*GFP* controls; the scale bars in E represent 1000 μm; gut transcriptional response of *BdCncC*, *BdMafK*, and *BdKeap1* to *BdNOX5* silencing in (F) the RS and (G) RS + axenic strain flies; the mRNA levels of the P450 and GST genes in the guts of the *BdNOX5* knockdown (H) RS and (I) RS + axenic flies; P450 activity in the gut of (J) the RS and (K) RS + axenic flies after *BdNOX5* knockdown; GST activity in the gut of (L) the RS and (M) RS *+* axenic flies after *BdNOX5* knockdown; RS + axenic strain: RS strain fed mixed antibiotic and sugar water solution for 4 days; RS + axenic+*LL/EC* strain: RS + axenic strain flies fed aseptic water for 24 h followed by continuous feeding of a 5 OD bacterial solution of *L. lactis* or *E. casseliflavus* for 3 days; ds*BdNOX5* + *LL*: *a*fter injection of 2 μg dsRNA into the RS + axenic strain, continuous feeding of 5 OD *L. lactis* was performed for 72 h; Conv, conventionally colonized flies; Axenic, axenic flies; Student’s *t-*test was performed for A–D and F–M; error bars indicate ±s.e.m. ^***^P < .001, ^**^P < .01, ^*^P < .05; all results were obtained from at least two independent experiments.

The production of ROS induced by the gut microbiota occurs mainly through the NADPH family enzymes *DUOX* and *NOX*. We measured the expression levels of *BdDUOX* and *BdNOX5* in the guts of SS and RS flies. Quantitative PCR revealed that the expression level of *BdNOX5* in the gut of the RS flies was significantly greater than that in the gut of the SS flies (Fig. 6A), whereas there was no difference in *BdDUOX* expression (Fig. S9A). Additionally, upon elimination of gut bacteria from the RS flies, the expression level of *BdNOX5* significantly decreased, and single bacterial supplementation with *L. lactis* or *E. casseliflavus* restored the expression of *BdNOX5* (Fig. 6B). Conversely, *K. pneumoniae* and *E. hormaechei*, which do not increase insecticide resistance in *B. dorsalis*, failed to activate highly expressed *BdNOX5* (Fig. S9B). Similar to those of the P450 and GST genes, the expression level of *BdNOX5* significantly increased following continuous oral administration of *L. lactis* at 0.5 OD and 5 OD for 3 days in the SS flies (Fig. S9C).

To validate the role of *BdNOX5* in the control of insecticide resistance in RS flies, we silenced *BdNOX5* expression by dsRNA injection. The interference efficiency of the ds-*BdNOX5* injection at 72 h was 57.1% in the traditionally fed RS and 27.3% in the axenic RS flies (Fig. 6C). After *BdNOX5* gene expression was significantly downregulated, the intestinal H_2_O_2_ content and ROS activity of the RS flies injected with ds-*BdNOX5* were significantly reduced (Fig. 6D and E). With ds-*GFP* as a control, the expression levels of the transcription factors *BdCncC* and *BdMafK*, which are regulated by ROS, in the intestines of both RS and axenic RS flies injected with ds-*BdNOX5* were significantly reduced, and the expression levels of *BdKeap1* were significantly increased (Fig. 6F and G). The expression of P450- and GST-related genes restricted by *BdCncC/MafK* transcriptional regulation also decreased significantly (Fig. 6H and I). Moreover, the P450 and GST enzyme activities decreased significantly (Fig. 6J–M). Furthermore, feeding *L. lactis* after injecting ds-*BdNOX5* into axenic flies did not reverse the reduction in H_2_O_2_ levels or ROS activity (Fig. 6D and E). Additionally, the low expression of *BdNOX5* did not result in activation of the *BdCncC* pathway, as indicated by the low expression levels observed (Fig. 6G). This inhibition, in turn, affected the high expression of detoxification genes (Fig. 6I) and the activities of the P450 and GST enzymes (Fig. 6K and M). In another interference experiment involving off-target control with *BdNOX5*-RNAi, the levels of the detoxification enzymes P450 and GST in the samples at 72 h also exhibited similar changes (Fig. S10). The data indicate that the gut microbiota regulates insecticide resistance by activating *BdNOX5*-ROS, which subsequently controls the expression and enzymatic activity of *BdCncC*-associated P450 and GST genes.

### LA production by the gut microbiota regulates detoxification

**Figure 7:**
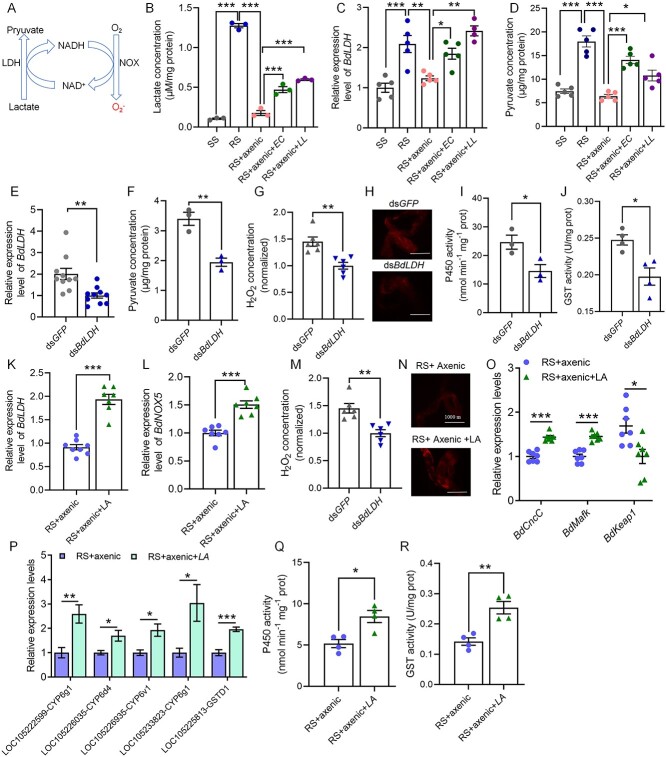
Lactate regulates detoxification in *B. dorsalis* through *BdLDH*; (A) scheme illustrating the potential mechanism of lactate-induced ROS generation in *D. melanogaster*; (B) levels of lactate in the guts of the SS, RS, RS + axenic, RS + axenic+*LL*, and RS + axenic+*EC* flies; *N* = 20 guts per biological duplication; (C) RT–qPCR showing LOC105231405-LDH expression in the guts of the SS, RS, RS + axenic, RS + axenic+*LL*, and RS + axenic+*EC* flies; (D) PA levels were measured in the guts of flies under different conditions: SS, RS, RS + axenic, RS + axenic+*LL*, and RS + axenic+*EC*; *N* = 30 guts per biological replicate; (E) *LOC105231405-LDH* silencing efficiency in *B. dorsalis* at 72 h postinjection of 2.0 μg of ds-*GFP* or ds-*BdLDH*-2 in the gut of RS strain flies; the data were normalized to the expression levels in the ds-*BdLDH*-2-treated flies; (F) PA concentrations were evaluated in the gastrointestinal tracts of the ds-*GFP-* and ds-*BdLDH*-2-treated flies after 72 h. ROS activity in the gut of the ds-*GFP-* and ds-*BdLDH*-2-treated flies after 72 h was measured by (G) H_2_O_2_ assays and (H) DHE analysis; (I) P450 and (J) GST activity in the guts of the ds-*GFP* and ds-*BdLDH*-2 flies after 72 h; RT–qPCR showing (K) *LOC105231405-LDH* and (L) *BdNOX5* expression in the guts of the flies of the indicated genotypes after 50 μM LA feeding for 72 h in the RS + axenic flies; ROS activity in the guts of the RS + axenic and RS + axenic+LA flies was measured by (M) H_2_O_2_ assays and (N) DHE analysis; (O) mRNA levels of the *BdCncC*, *BdMafK*, *BdKeap1*, (P) P450 and GST genes in the guts of the RS + axenic and RS + axenic+LA flies; (Q) P450 activity and (R) GST activity in the gut of the RS + axenic and RS + axenic+LA flies; the H_2_O_2_ levels in G and M were normalized to those in the RS + axenic controls; the scale bars in H and N represent 1000 μm; Student’s *t*-test was performed for B–G, I–M, and O–R; error bars indicate ± s.e.m.; ^***^P < .001, ^**^P < .01, ^*^P < .05; all results were obtained from at least two independent experiments.

*DUOX*-dependent ROS are induced by gut microbe-derived uracil [41], whereas *NOX*-dependent ROS are induced by LA produced by LA-producing bacteria [42]. *L. lactis* and *E. casseliflavus* are representative LA bacteria (LAB) that frequently acidify substrates via the release of large amounts of LA [43–46]. LA in the intestine is converted to pyruvic acid by lactate dehydrogenase (LDH), and simultaneously, NAD^+^ is reduced to NADPH; then, NADPH is converted to NAD^+^ and superoxide (O^2−^) via NADPH oxidase [42, 47, 48] (Fig. 7A).

In our study, the intestinal lactate content in the RS group was significantly greater than that in the SS group (Fig. 7B). After intestinal bacteria were eliminated with antibiotics, the lactate content also decreased significantly, and the lactate content in the intestine increased after single bacterial supplementation with *L. lactis* or *E. casseliflavus* (Fig. 7B). We screened two LDH genes in the genome of *B. dorsalis*: *LOC105231405-LDH* and *LOC105231406-LDH*. The quantitative PCR results showed no difference in the expression of *LOC105231406-LDH* in the guts of the SS and RS flies (Fig. S11). However, compared with that in the SS intestine, *LOC105231405-LDH* was significantly overexpressed in the RS intestine but was downregulated after the elimination of intestinal bacteria (Fig. 7C). Single bacterial supplementation with *L. lactis* or *E. casseliflavus* restored the high expression of *LOC105231405-LDH* (Fig. 7C). Moreover, the increase in lactate levels and the high expression of *LOC105231405-LDH* contributed to the increase in PA content (Fig. 7D). Furthermore, we effectively inhibited *LOC105231405-LDH* at 48 h and 72 h after injection of ds-*BdLDH*-1 and ds-*BdLDH*-2 (Fig. S12A). The interference efficiency of ds-*BdLDH*-2 at 72 h reached 50.1% (Fig. 7E). Additionally, the knockdown of *LOC105231405-LDH* significantly reduced the levels of PA in the gut of the RS flies (Fig. 7F), the ROS content (Fig. 7G and H), and the enzyme activities of P450 (Fig. 7I and Fig. S12B) and GST (Fig. 7J and Fig. S12C).

After continuously feeding SS flies with different concentrations of LA for 72 h, we found that both 5 μM and 50 μM LA increased the expression of *LOC105231405-LDH*. However, 500 μM and 50 mM LA restored the expression of *LOC105231405-LDH* (Fig. S13A). Moreover, feeding 50 μM LA to axenic RS flies led to elevated *LOC105231405-LDH* expression at 48 h (Fig. 7K). LA feeding after antibiotic treatment activated *BdNOX5* expression (Fig. 7L) and ROS activity (Fig. 7M and N), replacing *L. lactis* and *E. casseliflavus*. Continuous LA feeding in the SS flies also activated *BdNOX5* expression (Fig. S13B) and ROS activity (Fig. S13C). LA-induced ROS activated the *BdCncC* pathway (Fig. 7O), regulating P450 and GST expression (Fig. 7P) and increasing their enzyme activities (Fig. 7Q and R). Higher P450 and GST enzyme activities were also observed in the SS LA feeding experiment (Fig. S13D and E). These findings identify a central role for *LOC105231405-LDH* in ROS generation by *L. lactis* and *E. casseliflavus*, likely by oxidizing lactate and generating PA and NADH, a substrate for *BdNOX5*.

## Discussion

Numerous studies have shown that symbiotic bacteria of the insect gut regulate the activity of detoxifying enzymes involved in metabolism of host food sources, thereby indirectly increasing host resistance [8–11]. Although symbiotic bacteria and insect resistance are linked, the physiological and biochemical mechanisms underlying this relationship have not been systematically elucidated. In our study, the gene expression and enzyme activity of P450 and GST in the gut were significantly greater in the RS flies that were chronically exposed to β-cypermethrin, which is a broad-spectrum pyrethroid insecticide, than in the SS flies. We demonstrated that the LAB *L. lactis* and *E. casseliflavus* were enriched in the gut of the RS flies. Further experiments revealed that ROS generated by LAB activated the *BdCncC*/*BdKeap1*/*BdMafK* pathway, which modulates downstream P450 and GST activities and increases insecticide resistance in *B. dorsalis*. Therefore, our current findings elucidate the pathway by which the gut microbiota indirectly regulates host resistance to insecticides.

Mutually beneficial symbiotic insect–bacteria relationships help symbiotic systems adapt to pesticide screening pressures and allow continued coevolution [49]. Thus, the increased resistance to insecticides in our RS flies may be attributed to coevolution with LAB. *Enterococcus* has been shown to increase insecticide tolerance in *Spodoptera litura* [50], *B. mori* [51], *Plutella xylostella* [52], and *Spodoptera frugiperda* [53]. Genomic analysis of strains of *Enterococcus* isolated from *S. frugiperda* larvae revealed that they possessed exogenous degradation enzyme mechanisms [53]. When honeybees (*Apis mellifera*) and *D. melanogaster* are exposed to insecticides, the relative abundance of LAB typically increases [32, 54–56]. In this study, the activities of P450 and GST were inhibited after feeding with mixed antibiotic solutions to clear the intestinal commensal bacteria in *B. dorsalis*. In contrast, oral administration of *L. lactis* or *E. casseliflavus* rescued the activities of P450 and GST and increased the tolerance of the flies to β-cypermethrin. We demonstrated that detoxification does not occur through direct encoding of hydrolases but rather through modulation of the host’s physiological and biochemical environment, thereby indirectly increasing the detoxification capacity of the host via intracellular signaling pathways. This regulatory activity is dependent on ROS levels in the gut of *B. dorsalis*. We found here that the induction of ROS by LAB required *BdNOX5* but not *BdDUOX*. However, *DUOX* is mainly activated by uracil secreted by pathogenic bacteria and some symbiotic gut bacteria [57]. We identified *L. lactis-* and *E. casseliflavus*-derived LA as an essential metabolite triggering NOX5-dependent ROS production. Similar results have been obtained in *Drosophila*, in which LA secreted by *L. plantarum* can drive ROS production via *NOX* [42]. Moreover, the decrease in the abundance of *Vagococus*, *Commesalibacter*, *Stenotrophomonas*, *E. Shigella*, and *Providencia* in the gut of the RS group may be attributed to the increase in ROS, which are important immune factors in the gut [58].

Most previous studies have concluded that insect resistance mechanisms are mainly categorized into metabolic resistance and target resistance [59, 60]. The detoxification enzymes most closely associated with metabolic resistance include carboxylesterases [61], P450s [62, 63], GSTs [64], and aldehyde oxidase [65, 66]. The transcription factor CncC is a central regulatory factor in the response of insects to xenobiotics, and its actions include induction of the expression of the detoxification genes P450 and GST [12]. ROS interact with key signaling molecules to initiate signaling in various cellular processes and play an important role in cellular signaling cascades [67]. Previously, numerous studies have demonstrated that chemical pesticide-induced ROS bursts activate detoxification activities related to the *CncC/Keap1/Maf* pathway [12, 13, 37]. For example, exposure to clothianidin in *Bradysia odoriphaga* caused ROS accumulation that activated the CncC pathway and involved the P450 genes [68], and the upregulation of resistance genes induced by indoxacarb exposure in *S. litura* was also predicted to involve a CncC/Maf binding site [37]. Moreover, xanthotoxin exposure increased ROS levels and activated the expression of the *CncC/MafK* pathway and detoxification genes in the larvae of *S. litura*, which in turn increased tolerance to λ-cypermethrin [69]. In our study, the detoxification genes that varied with the level of ROS produced by enteric bacteria were all predicted to have CncC/Maf binding sites. VC feeding experiments showed that changes in ROS levels induced by *L. lactis* and *E. casseliflavus* in *B. dorsalis* also led to corresponding regulation of the *BdCncC*/*BdKeap1*/*BdMafK* pathway and the expression of downstream detoxification genes, as well as enzyme activity. Furthermore, the associated detoxification activity induced by symbiotic bacteria was inhibited after *BdCncC* knockdown, indicating that *BdCncC* is indispensable for activating the associated detoxification enzymes due to changes in the intestinal physicochemical environment caused by symbiotic bacteria. These results provide a theoretical basis for the indirect regulation of insect resistance by symbiotic bacteria.

The degree of resistance decreased as the bacterial concentration increased to 10 OD. Likewise, at concentrations greater than 500 μM, LA also reduced ROS activation in the gut. This observation suggests a potential self-protective mechanism of the organism. Excessive ROS cause oxidative stress damage to intestinal epithelial cells, resulting in irreversible damage to cellular components and leading to cell death [70]. In the *Drosophila* intestine, when the immune deficiency pathway, which produces various antimicrobial peptides to regulate the balance of the intestinal microbiota, is blocked, the substantial proliferation and accumulation of *L. plantarum* lead to an excess of ROS, which ultimately damages intestinal epithelial cells and accelerates their death [42]. In addition, when many exogenous pathogens invade the intestinal tract, *DUOX* regulates ROS, scavenges overexpressed ROS via the immune response through a variety of ROS-scavenging enzymes, such as SOD, CAT, and POD, and maintains ROS levels within the threshold for host damage [58]. However, ROS are also central regulatory factors for intestinal stem cell (ISC) renewal. When spatiotemporally limited, ROS initiate Jnk signaling and activate calcineurin/CRTC and ERK signaling, as well as the JAK/STAT signaling pathway, leading to downstream ISC-induced intestinal epithelial cell renewal [71].

In summary, our research provides a deeper understanding of the molecular mechanisms by which the insect gut microbiota regulates insecticide resistance through indirect physiological and biochemical reactions. For more effective pest control, innovative control strategies such as the use of nanoantibiotics [72] carrying insecticides can be developed to address the challenge of increasing insecticide resistance by symbiotic bacteria. Furthermore, the screened P450 and GST genes can serve as potential target sites for overcoming insecticide resistance.

## Supplementary Material

supportong_information_wrae058

Supplementary_Table_wrae058

## Data Availability

All the data needed to evaluate the conclusions in the paper are presented in the paper and/or the Supplementary Materials. The raw transcriptome and 16S rRNA gene sequencing data were deposited in the NCBI SRA under the accession numbers PRJNA1027602 and PRJNA1027561.
