# Supplementary material for: Lactic acid bacteria modulate the CncC pathway to enhance resistance to β-cypermethrin in the oriental fruit fly
Source: ISME J. 2024 Apr 15;18(1):wrae058. doi: 10.1093/ismejo/wrae058 (PMC11069359; doi:10.1093/ismejo/wrae058)
Supplement: supportong_information_wrae058 [file supportong_information_wrae058.docx]

**Lactic Acid Bacteria Modulate the CncC Pathway to Enhance Resistance to β-cypermethrin in the Oriental Fruit Fly**

Tian Zeng^1^, Qianyan Fu^1^, Fangyi Luo^2^, Jian Dai^1^, Rong Fu^1^, Yixiang Qi^1^, Xiaojuan Deng^2^, Yongyue Lu^1^, Yijuan Xu^1^*

^1^Guangdong Laboratory for Lingnan Modern Agriculture, Department of Entomology, South China Agricultural University, Guangzhou 510642, China

^2^Guangdong Prov Sericulture & Mulberry Engn Res Ct, Guangdong Prov Key Lab Agroanim Genom & Mol Breed, Coll Anim Sci, South China Agricultural University, Guangzhou 510642, China

*Correspondence to: Dr. Yijuan Xu (xuyijuan@yahoo.com), South China Agricultural University, Wushan Road 483, Tianhe District, Guangzhou 510642, China.

**Supporting information**

**
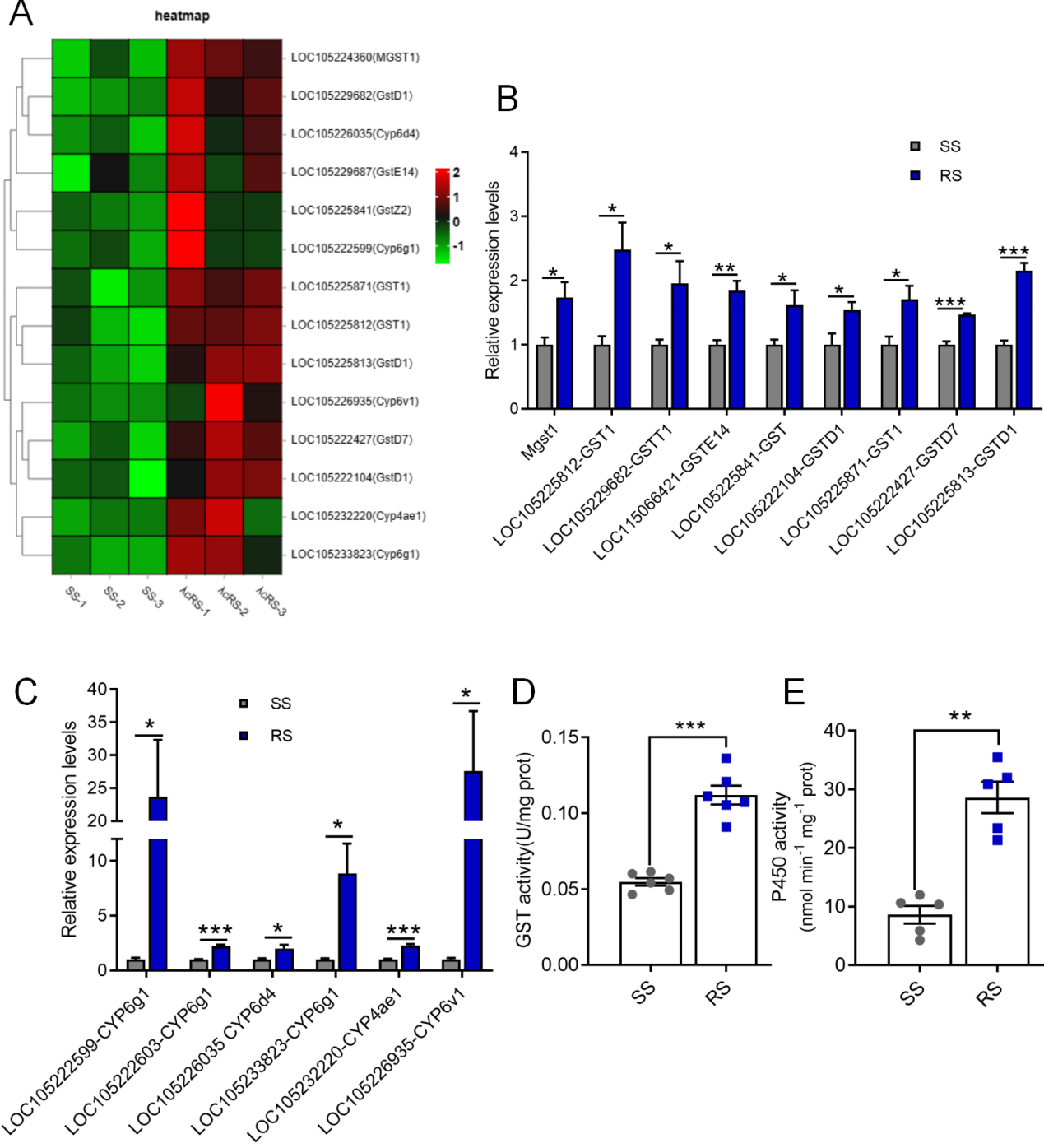
**

**Fig. S1.** Differences in P450 and GST gene expression between the RS and SS groups. (A) Heatmap showing the relative expression levels of genes involved in detoxification in the gut of *B. dorsalis*. The map is plotted based on log2-transformed FPKM values, and each bar or column corresponds to the relative expression level of the gene in one sample, with warmer colors representing higher relative gene expression levels. (B-C) qPCR validation of differentially expressed genes, including LOC105222599-CYP6g1, LOC105222603-CYP6g1, LOC105226035-CYP6d4, LOC105233823-CYP6g1, LOC105226935-CYP6v1, LOC105232220-CYP4ae1, LOC105224360-Mgst, LOC105225812-GST1, LOC105229682-GSTT1, LOC115066421-GSTE14, LOC105225841-GST, LOC105222104-GSTD1, LOC105225871-GST1, LOC105222427-GstD7, and LOC105225813-GSTD1, in the gut of the SS and RS flies. (D) P450 activity and (E) GST activity in the gut of *B. dorsalis* between the SS and RS flies. Student’s t test was performed for B-E. Error bars indicate ±s.e.m. ****p*<0.001, ***p*<0.01, **p*<0.05.


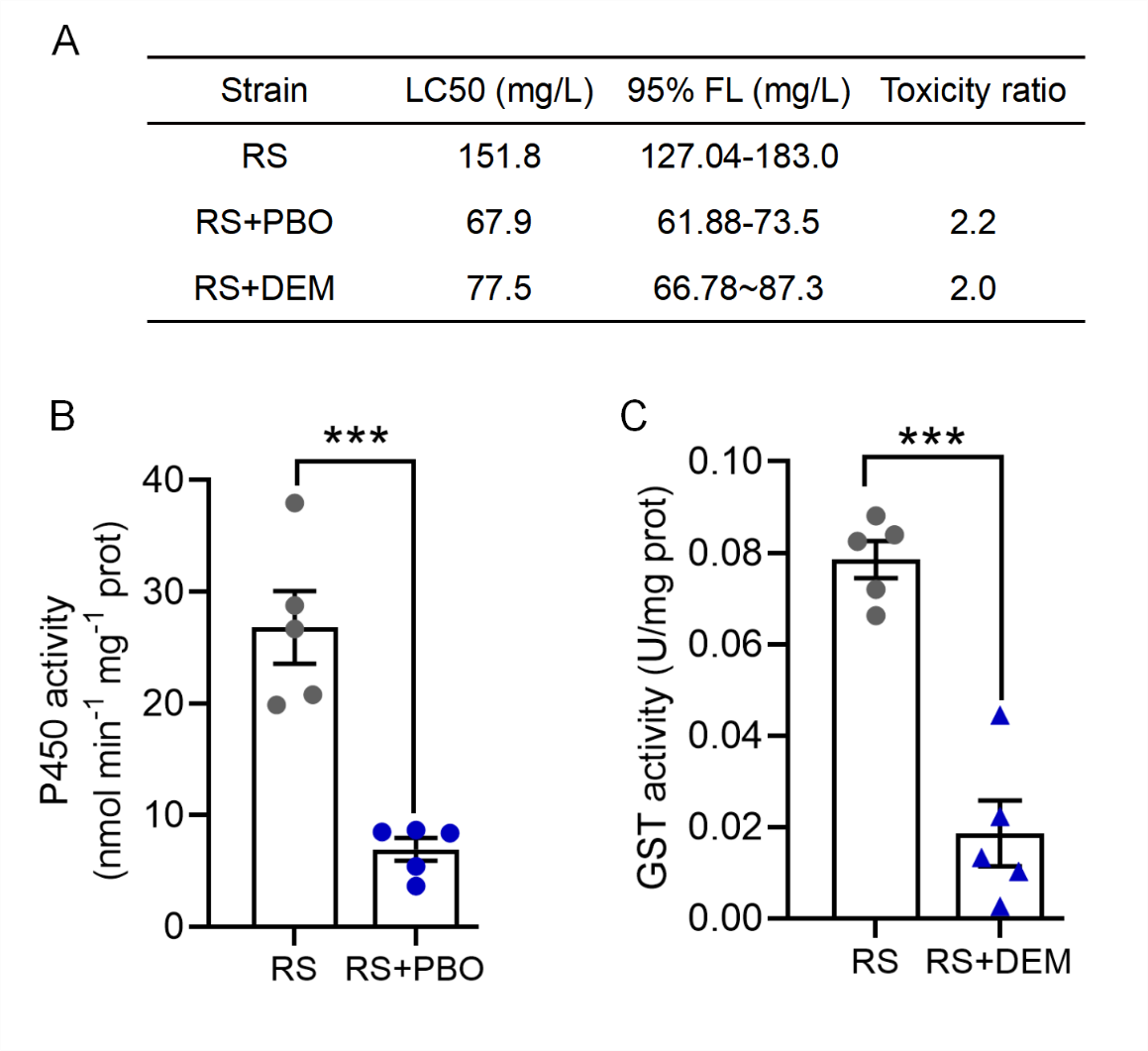


**Fig. S2.** Effects of PBO and DEM on β-cypermethrin toxicity in RS *B. dorsalis*. (A) Susceptibility of PBO- and DEM-treated RS flies to β-cypermethrin. (B) P450 activity and (C) GST activity in the gut of *B. dorsalis* between the SS and RS+PBO or RS+DEM groups. The toxicity ratio was calculated as the LC_50_ of the RS to that of the RS+PBO or RS+DEM group. LC_50_ values were considered significantly different if their fiducial limits did not overlap. Student’s t test was performed for B and C. Error bars indicate ± s.e.m. ****p*<0.001, ***p*<0.01, **p*<0.05.

**
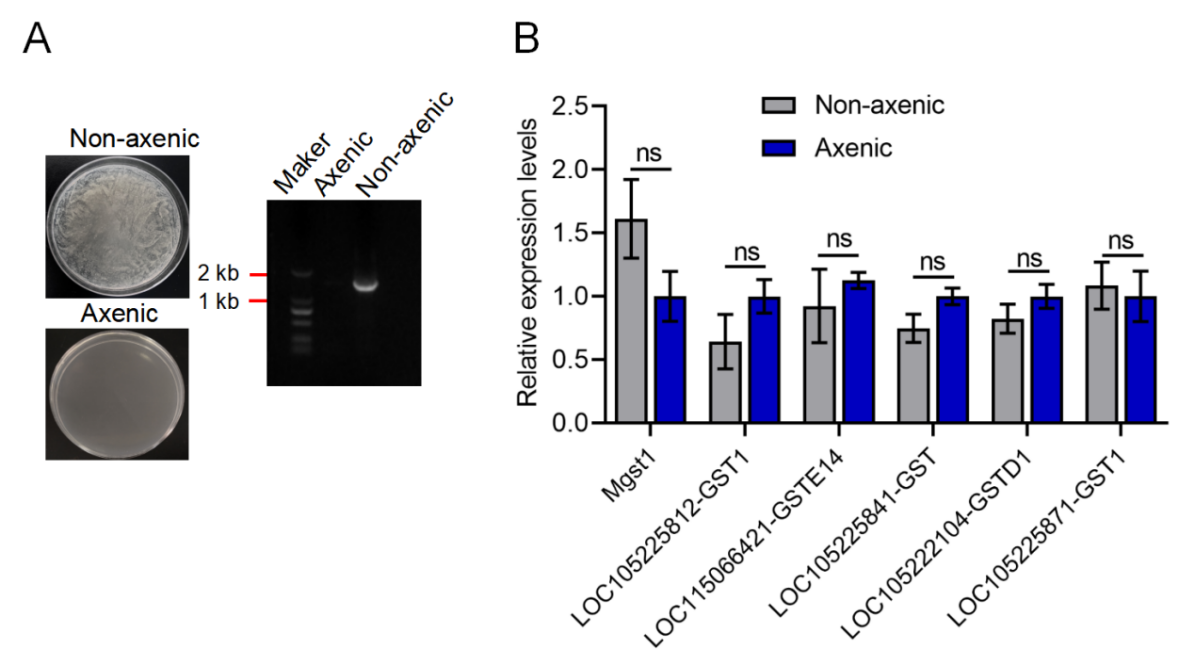
**

D

C

**Fig. S3.** Elimination of gut bacteria from the RS. (A) Efficacy of eliminating gut bacteria confirmed by culturing *B. dorsalis* gut homogenates on LB agar plates and by performing PCR analysis on gut homogenates using universal 16S rRNA gene primers. (B) Detoxification genes with no change in gut mRNA after removal of intestinal bacteria in the RS. The data were analyzed by Student’s t test. Error bars indicate ±s.e.m. ns indicates *p*>0.05.


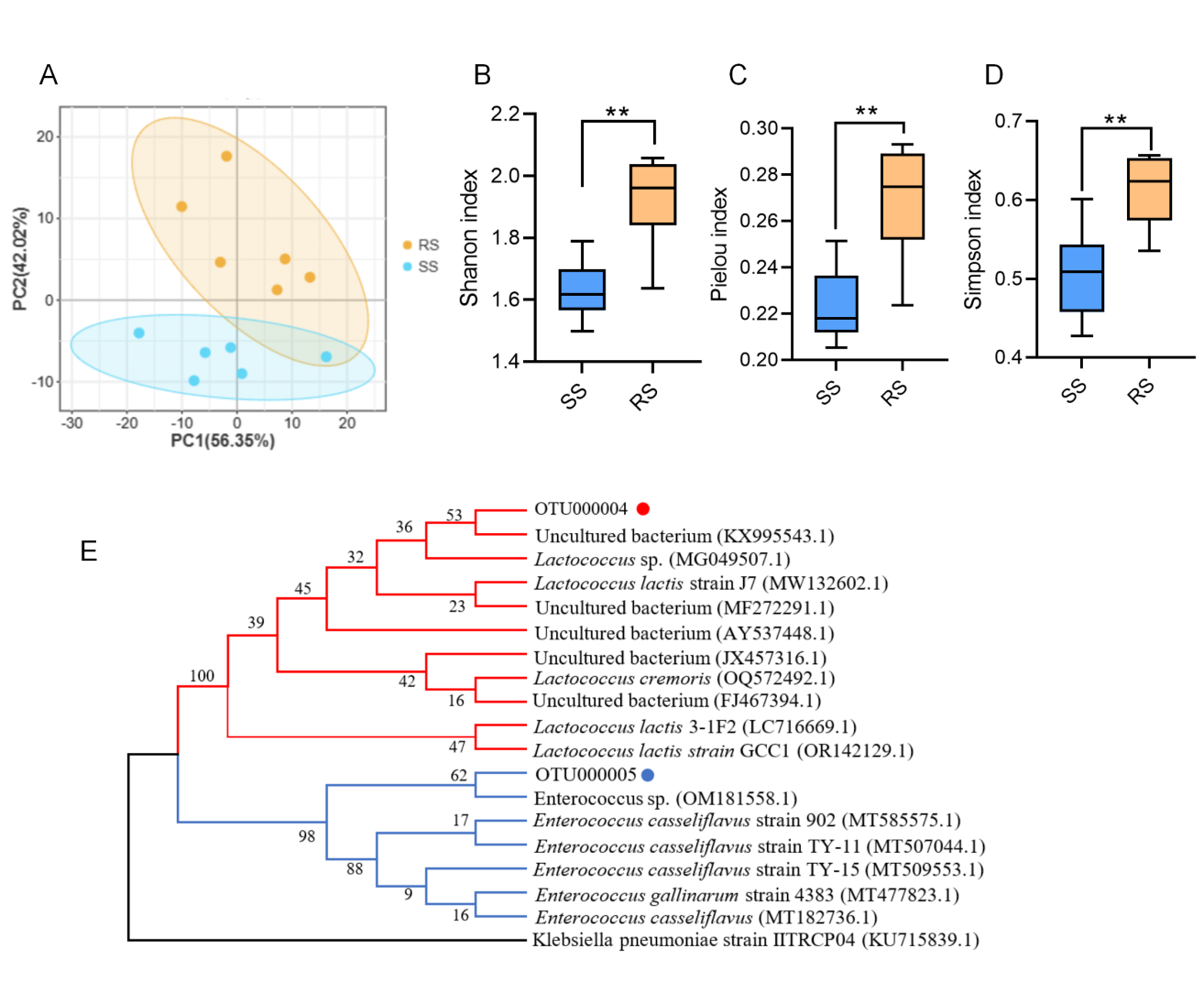


**Fig. S4.** Differences in the microbial composition of the gut between the SS and RS groups are structural. (A) PCA of unweighted jackknife UniFrac distances of gut microbial communities from the SS and RS flies. Each symbol represents a sample. (B-D) Richness measured by the Shannon, Pielou, and Shannon indices of gut bacterial communities from the SS and RS flies. (E) Phylogenetic analysis of the identified *E. casseliflavus* and *L. lactis* operational taxonomic units (OTUs) with 16 related bacteria. The data were analyzed by Student's t test for B-D. Error bars indicate ±s.e.m. ****p*<0.001, ***p*<0.01, **p*<0.05.

b

a

**
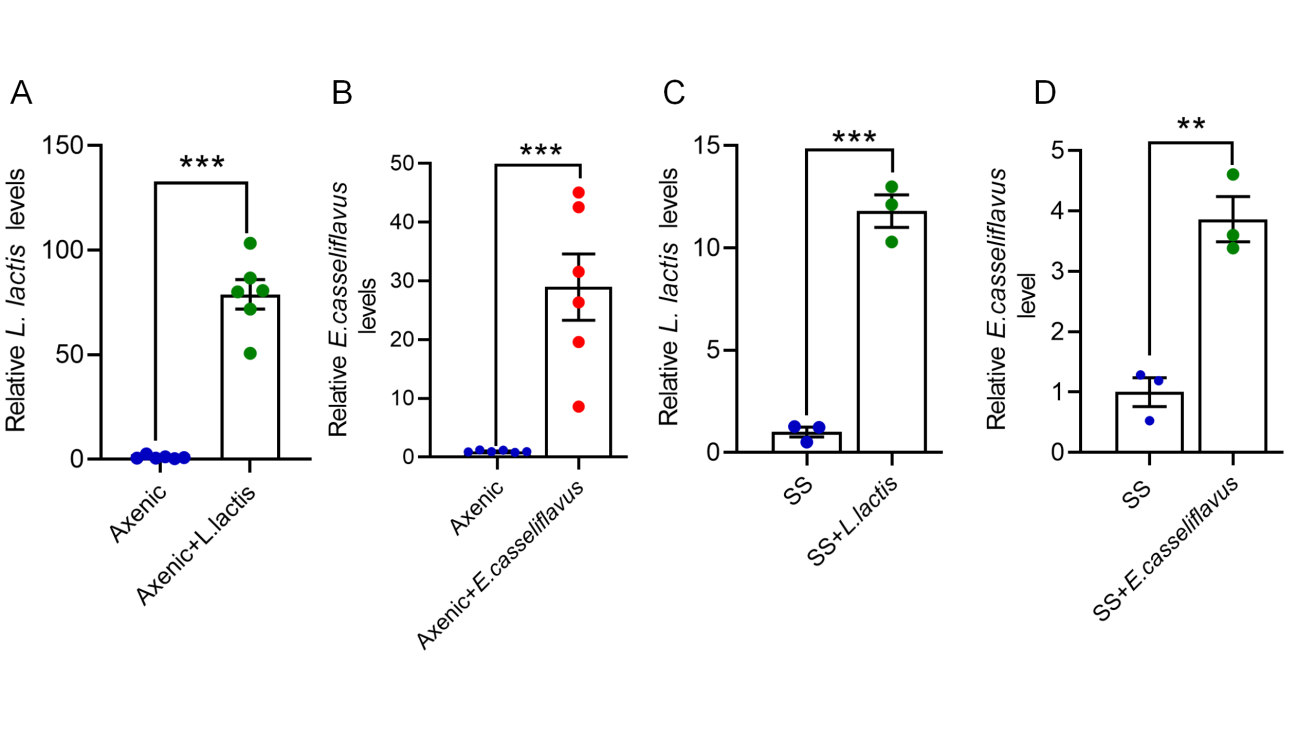
Fig. S5.** Confirmation via single bacterial refeeding after antibiotic treatment. Intestinal bacterial DNA qPCR validation for specific bacteria: (A) *L. lactis* in the RS+axenic and RS+axenic+*L. lactis* flies and (B) *E. casseliflavus* in the RS+axenic and RS+axenic+*E. casseliflavus* flies*.* RS+axenic flies: continuously fed mixed antibiotics; RS+axenic+*L. lactis/E .casseliflavus*: after 4 days of continuous feeding with mixed antibiotics and 1 day of aseptic water treatment, feeding for 3 days with 5 OD 8% sugar water and resuspended bacterial solution was performed. (C-D) qPCR validation of (C) *L. lactis* or (D) *E. casseliflavus* in SS flies after feeding with 5 OD 8% sugar water and resuspension of the bacterial solution for 3 days. The data were analyzed by Student’s t test. Error bars indicate ±s.e.m. ****p*<0.001, ***p*<0.01, **p*<0.05.

A B


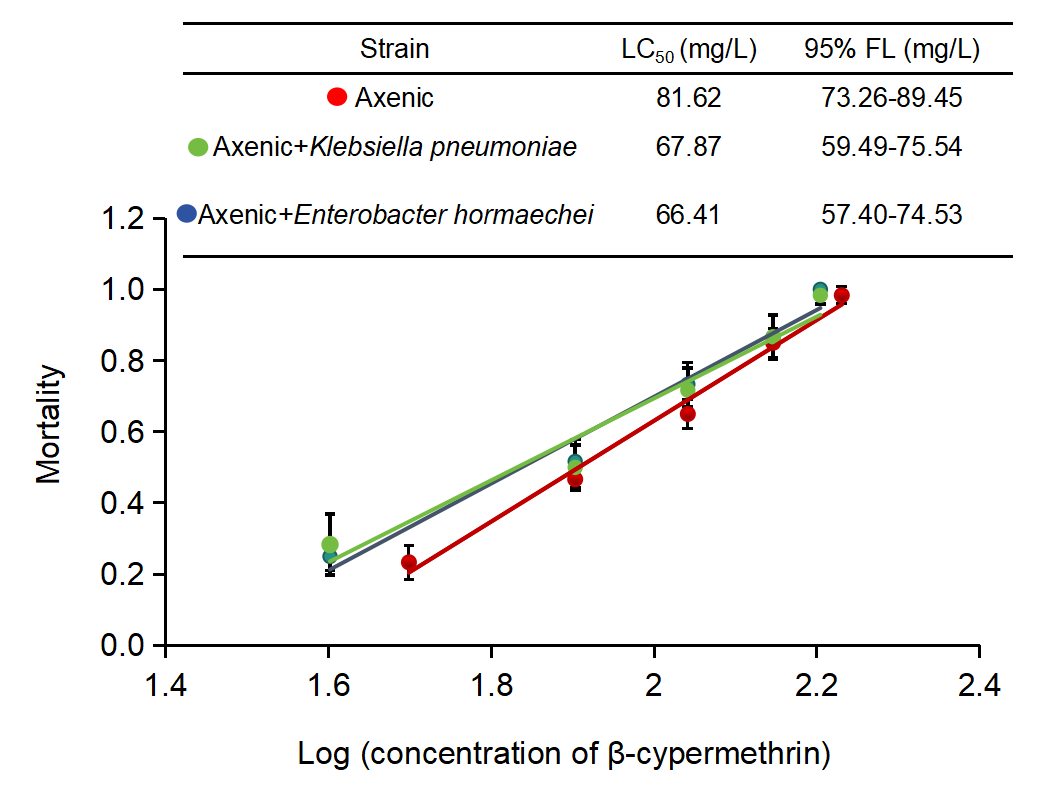

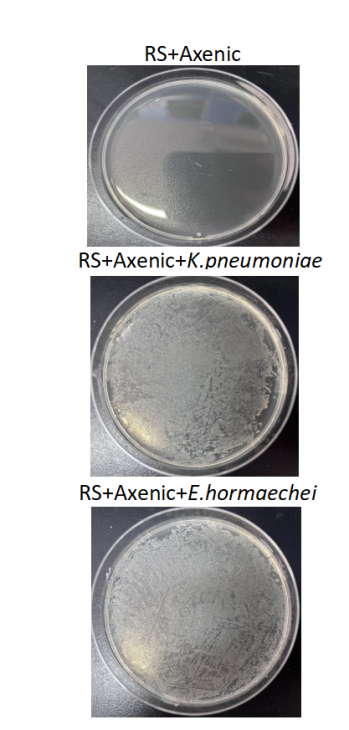


**Fig. S6.** (A) Intestinal bacterial CFU validation for the RS+axenic, RS+axenic+*K. pneumoniae* and RS+axenic+*E. hormaechei* flies*.* (B) Susceptibility to β-cypermethrin in the RS+axenic, RS+axenic+*K. pneumoniae* and RS+axenic+*E. hormaechei* flies*.* LC_50_ values were considered significantly different if their fiducial limits did not overlap.


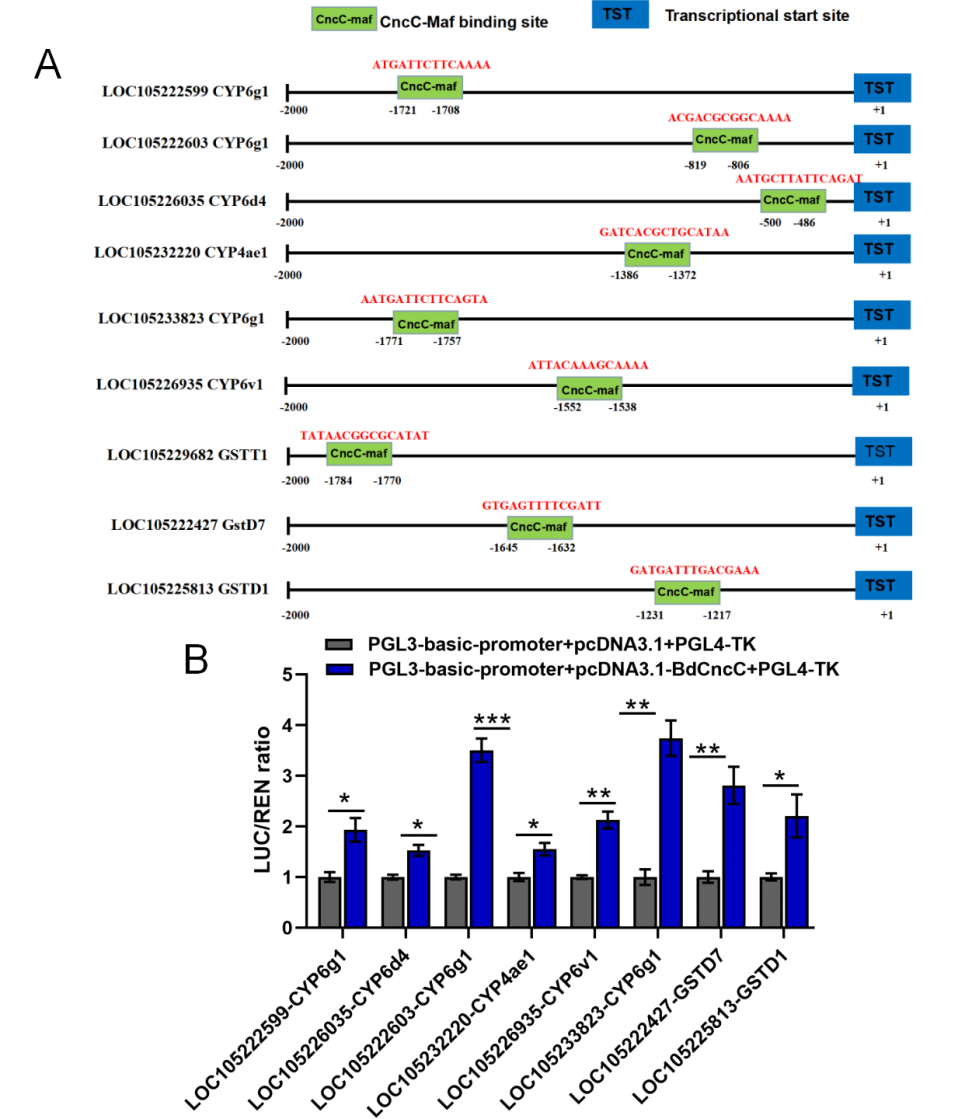


**Fig. S7.** The transcription factor CncC initiates the expression of P450 and GST resistance genes. (A) Prediction of the CncC–Maf binding site in the promoter regions of β-cypermethrin resistance-related genes activated by *L. lactis* or *E. casseliflavus* in *B. dorsalis*. TST is the transcriptional start site and is indicated by +1. (B) Detection of the interaction between the transcription factor *BdCncC* and the promoter regions of P450 and GST genes using a dual-luciferase reporter gene assay. LUC: firefly luciferase, REN: Renilla luciferase. The data were analyzed by Student’s t test. Error bars indicate ±s.e.m. ****p*<0.001, ***p*<0.01, **p*<0.05, ns indicates *p*>0.05.


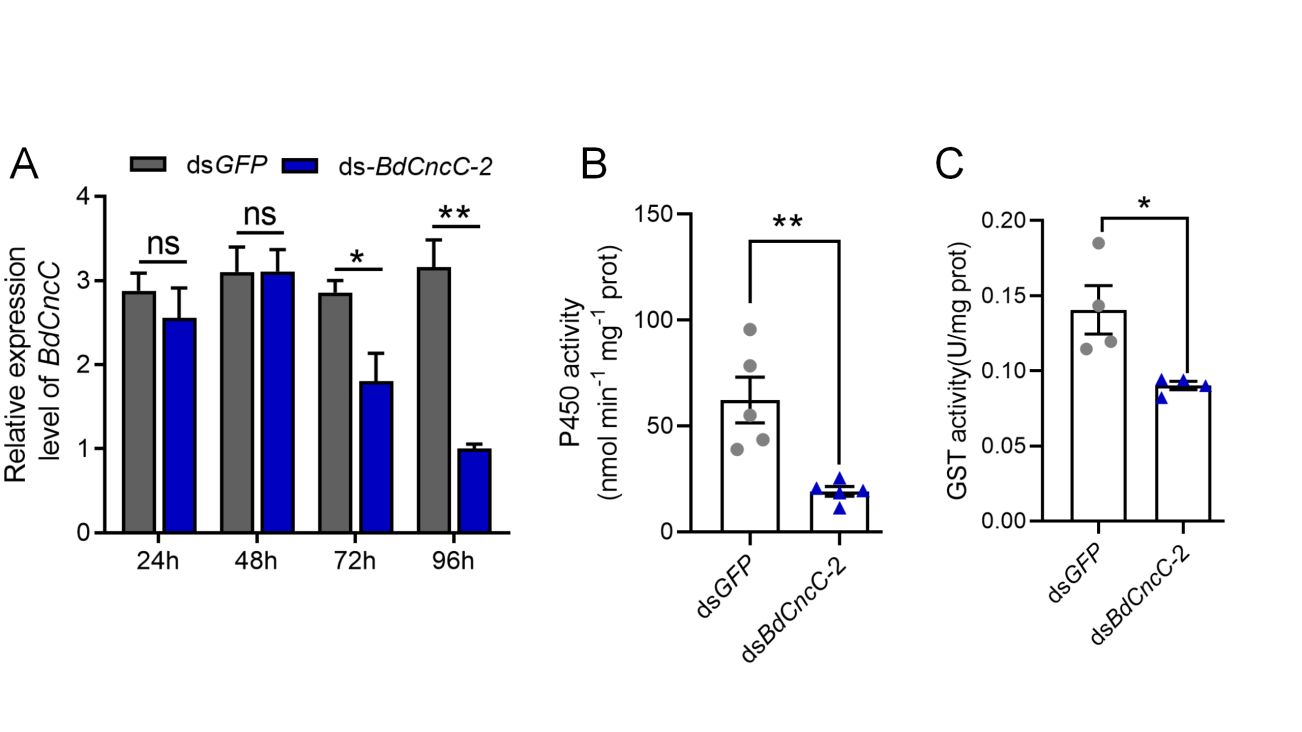


**Fig. S8.** Off-target detection of the *BdCncC* gene. (A) Interference efficiency at different time points after injecting 2 μg of ds-*BdCncC*-2, with ds-*GFP* as a control. Changes in (B) P450 and (C) GST enzyme activity 96 h after injecting 2 μg of ds-*BdCncC*-2. Student’s t test was performed. Error bars indicate ± s.e.m. ****p*<0.001, ***p*<0.01, **p*<0.05, ns indicates *p*>0.05.


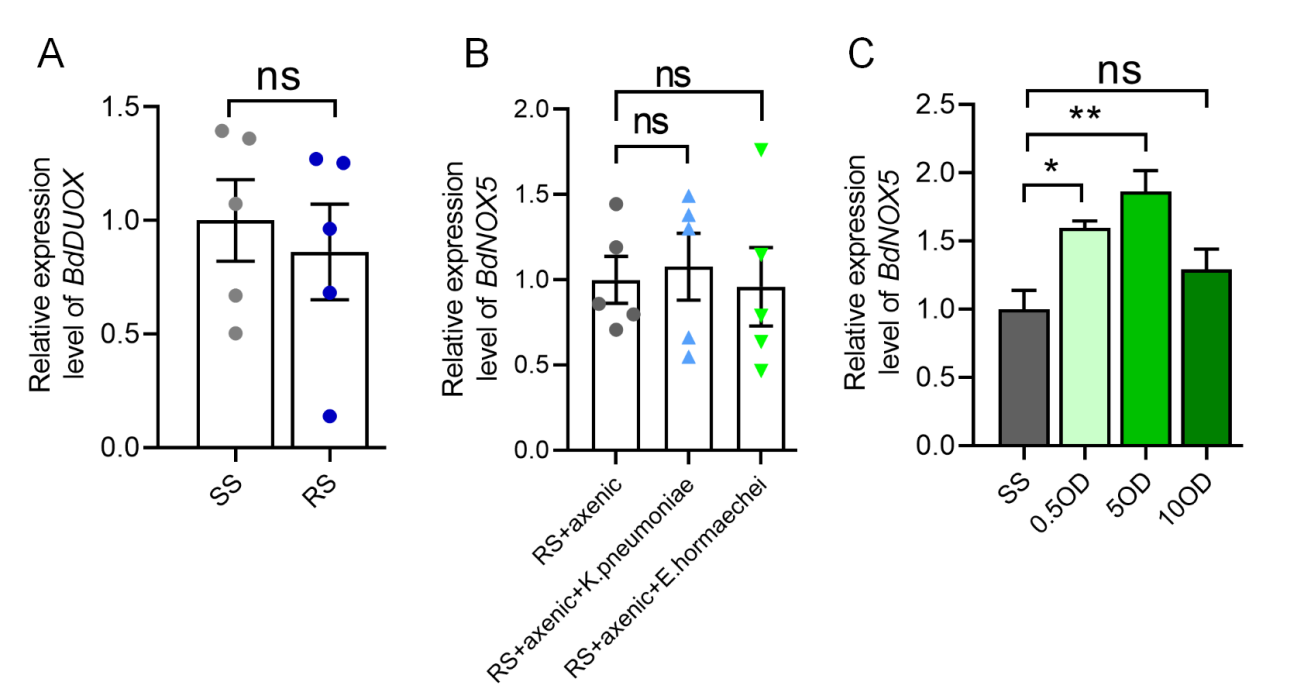


**Fig. S9.** Pattern of production of drug-activating ROS by intestinal commensal bacteria in *B. dorsalis*. (A) Gut mRNA expression of *BdDUOX* in the SS and RS flies. (B) Gut mRNA expression of *BdNOX5* in the RS+axenic, RS+axenic+*K.pneumoniae* and RS+axenic+*E.hormaechei* flies. (C) Gut mRNA expression of *BdNOX5* in the SS flies continuously fed different concentrations of *L. lactis* after 3 days. The data of A and B were analyzed by Student's t test, and the data of C were analyzed by one-way ANOVA followed by Tukey’s multiple comparison. Error bars indicate ±s.e.m. ****p*<0.001, ***p*<0.01, **p*<0.05, ns indicates *p*>0.05.


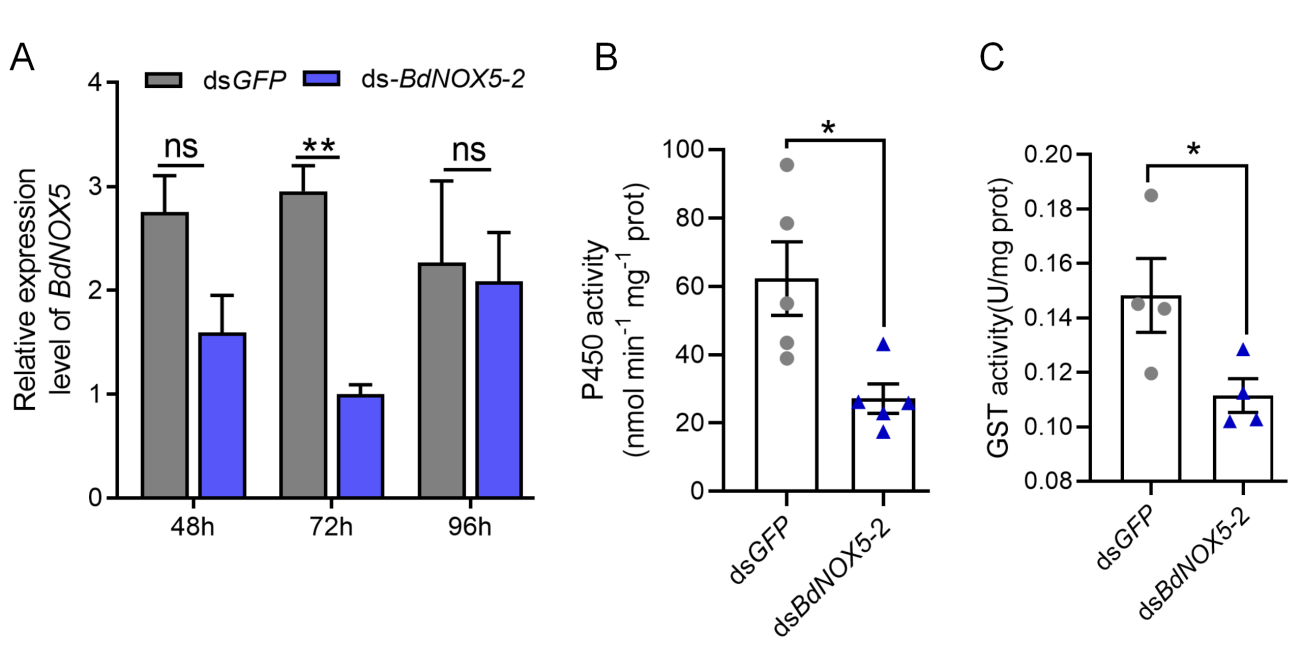


**Fig. S10.** Off-target analysis of the *BdNOX5* gene. (A) Interference efficiency at different time points after injecting 2 μg of ds-*BdNOX5*-2, with ds-*GFP* as a control. Changes in (B) P450 and (C) GST enzyme activity 72 h after injecting 2 μg of ds-*BdNOX5*-2. Student’s t test was performed. Error bars indicate ± s.e.m. ****p*<0.001, ***p*<0.01, **p*<0.05, ns indicates *p*>0.05.


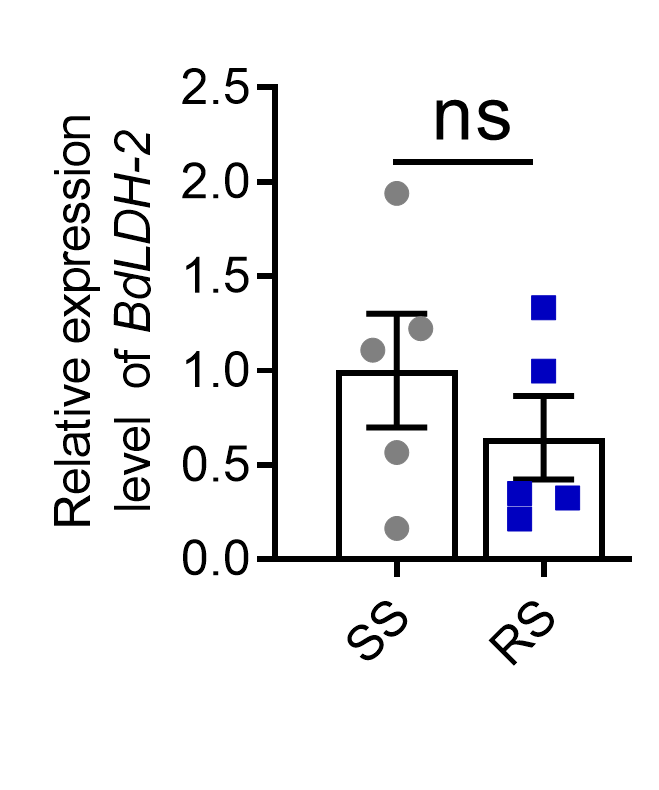


**Fig. S11.** RT‒qPCR showing LOC105231406-LDH expression in the guts of SS and RS flies. Data were analyzed by Student's t test. ns indicates *p*>0.05.


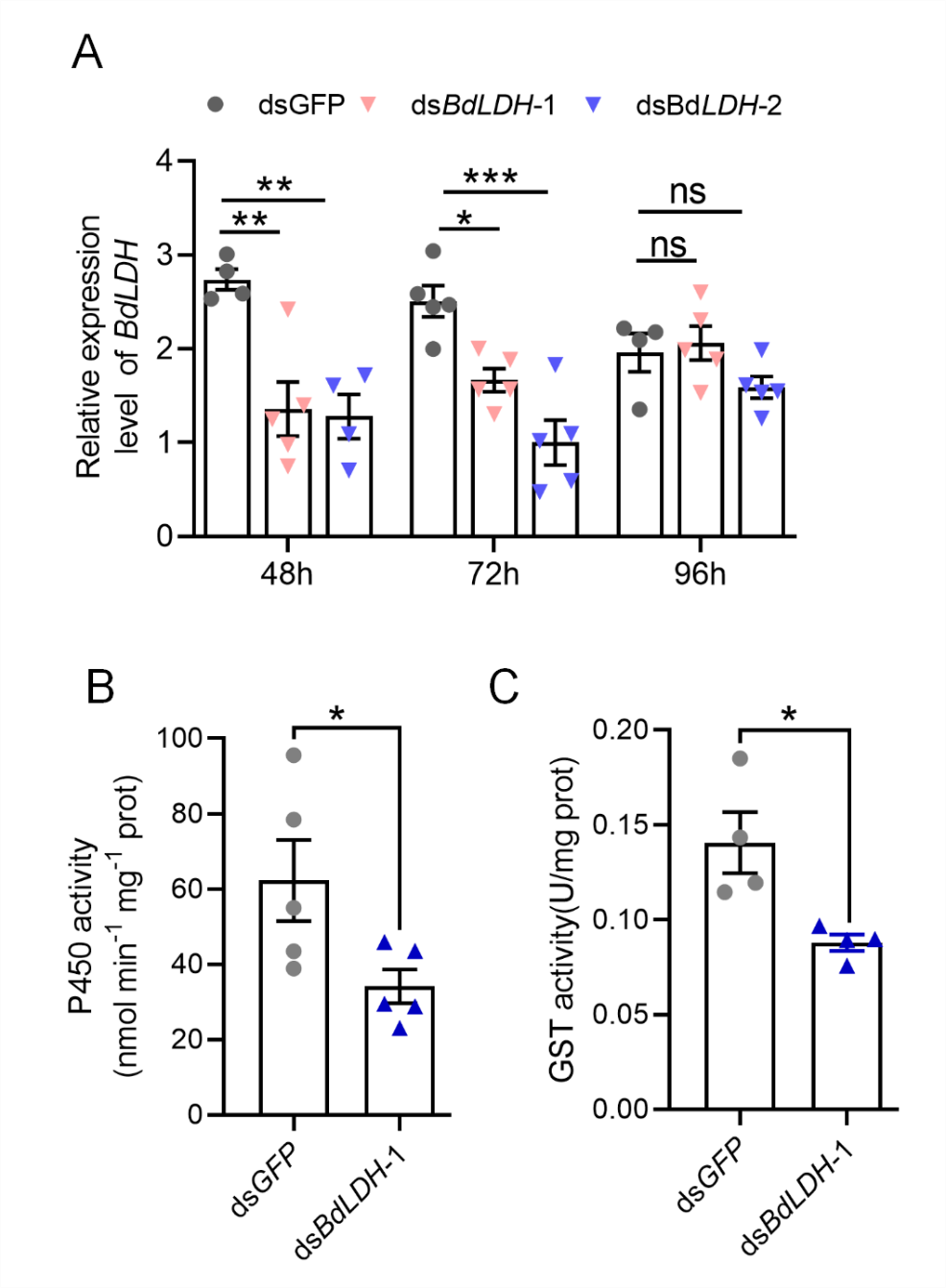


**Fig. S12.** Interference in LOC105231405-LDH gene expression in the RS strain. (A) The interference efficiency of the LOC105231405-LDH gene at different time points after injection of 2 μg of ds-*BdLDH*-1 or ds*BdLDH*-2 (with ds-*GFP* as a control). Changes in the enzymatic activity of (B) P450 and (C) GST in the intestinal tract of the RS flies 72 h after ds-*BdLDH*-1 injection. Student’s t test was performed. Error bars indicate ± s.e.m. ****p*<0.001, ***p*<0.01, **p*<0.05, ns indicates *p*>0.05.

**
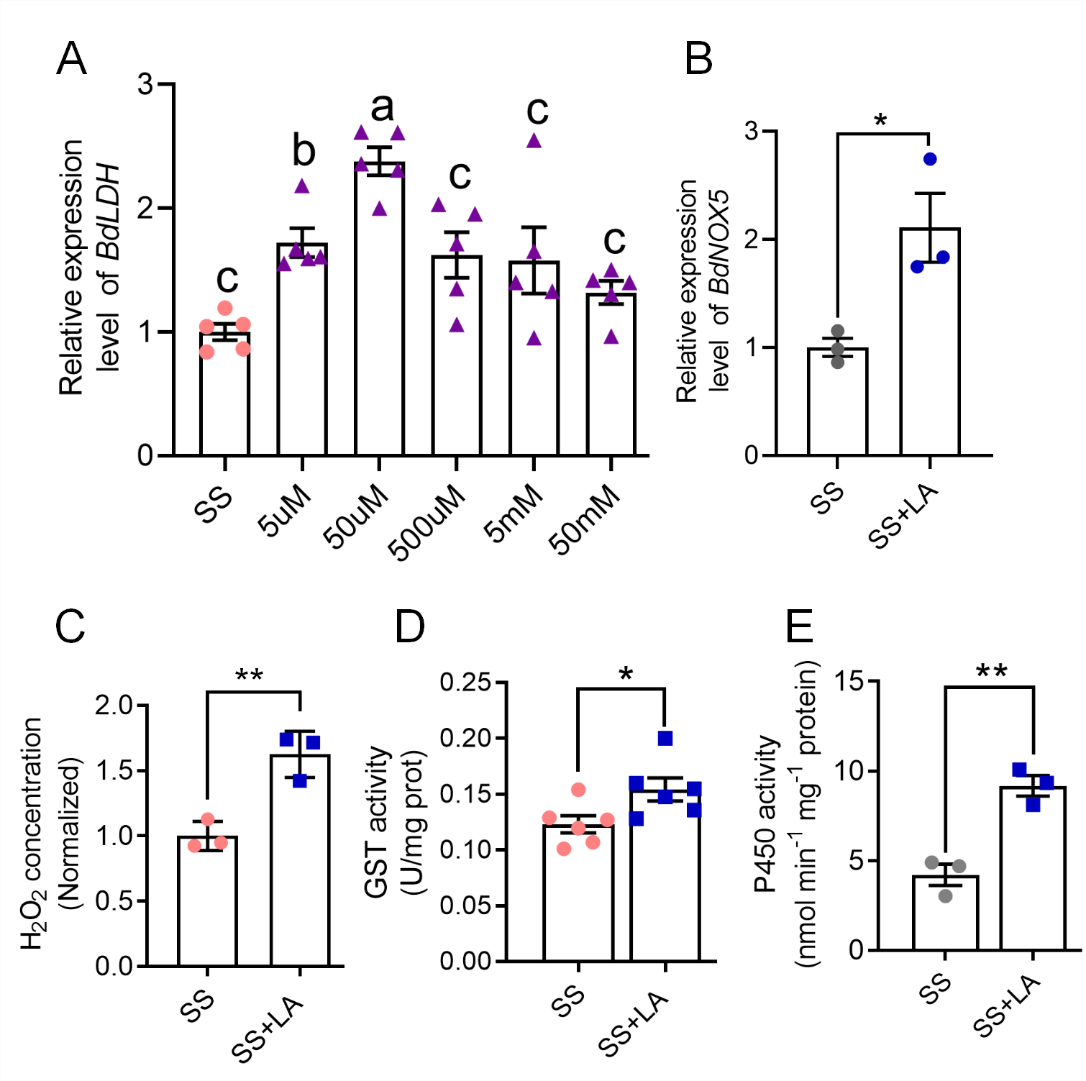
**

**Fig. S13.** The intestinal detoxification enzyme activity in the SS was increased by LA. (A) Gut mRNA expression of LOC105231405-LDH in SS flies continually fed different concentrations of lactate (LA) after 2 days. The error bars represent the standard error, and different letters above each bar indicate significant differences (*p*<0.05) according to one-way ANOVA followed by Tukey’s multiple comparison test. (B) Gut mRNA expression of *BdNOX5* in the SS and SS+LA flies. (C) ROS activity in the gut of the SS and SS+LA flies was measured by H_2_O_2_ assays. The H_2_O_2_ levels were normalized to those of the SS controls. (D) GST activity and (E) P450 activity in the gut of the SS and SS+LA flies. SS+LA: continuously fed 50 μM LA after 2 days. The data in B and D-G were analyzed by Student’s t test. The error bars indicate ±s.e.m. ****p*<0.001, ***p*<0.01, **p*<0.05.
